# Permissive and nonpermissive channel closings in CFTR revealed by a factor graph inference algorithm

**DOI:** 10.1016/j.bpr.2022.100083

**Published:** 2022-10-19

**Authors:** Alexander S. Moffett, Guiying Cui, Peter J. Thomas, William D. Hunt, Nael A. McCarty, Ryan S. Westafer, Andrew W. Eckford

**Affiliations:** 1 Department of Electrical Engineering and Computer Science, York University, Toronto, ON, Canada; 2 Emory + Children’s Center for Cystic Fibrosis and Airways Disease Research, Emory University School of Medicine and Children’s Healthcare of Atlanta, Atlanta, Georgia; 3 Department of Mathematics, Applied Mathematics, and Statistics, Case Western Reserve University, Cleveland, Ohio; 4 School of Electrical and Computer Engineering, Georgia Institute of Technology, Atlanta, Georgia; 5 Georgia Tech Research Institute, Atlanta, Georgia

## Abstract

The closing of the gated ion channel in the cystic fibrosis transmembrane conductance regulator can be categorized as nonpermissive to reopening, which involves the unbinding of ADP or ATP, or permissive, which does not. Identifying the type of closing is of interest as interactions with nucleotides can be affected in mutants or by introducing agonists. However, all closings are electrically silent and difficult to differentiate. For single-channel patch-clamp traces, we show that the type of the closing can be accurately determined by an inference algorithm implemented on a factor graph, which we demonstrate using both simulated and lab-obtained patch-clamp traces.

## Significance

Membrane ion channels are embedded in the plasma membranes of many eukaryotic cells, and the current through these channels can be measured using a patch-clamp apparatus. The opening and closing of an ion channel are dependent on a sequence of conformational changes between structural states, called kinetic microstates. These microstates are crucial to understanding the dynamics of the channel and are a subject of intense theoretical and experimental interest. In cystic fibrosis transmembrane conductance regulator, ATP binding occurs only in certain sequences of state transitions, while toxins, inhibitors, and point mutations are known to have a direct impact on these transitions. However, experiments to directly observe hidden (i.e., electrically equivalent) states are complex, and challenges remain in characterizing the states in their dynamic context.

In contrast to experimental methods, Bayesian inference and the expectation-maximization algorithm are simple and well-known techniques which have long been used as a solution to hidden-variable inference problems, including patch-clamp traces. These techniques are combined on graphical models called factor graphs. Applying a factor graph inference algorithm to cystic fibrosis transmembrane conductance regulator ion currents, our algorithm accurately distinguishes between permissive channel closings (without unbinding of nucleotide) and nonpermissive closings (with unbinding of nucleotide), providing insight into an otherwise hidden, but physiologically important, process. Our method is flexible and can be used to complement or improve other contemporary analytical or experimental techniques.

## Introduction

Cystic fibrosis (CF) is a life-threatening genetic disease affecting the respiratory and digestive systems that is caused by mutations to the CF transmembrane conductance regulator (CFTR) anion channel (1,2). CFTR is a “broken” member of the ATP-binding cassette transporter class in that CFTR acts as an ATP-gated ion channel rather than an active transporter as is the function of other ATP-binding cassette transporters. CFTR consists of a single polypeptide chain, with two transmembrane domain-nucleotide binding domain (NBD) pairs connected through a region called the R domain (3). Each of the two NBDs contribute to both of the two known binding sites for ATP, although only one of these sites facilitates the hydrolysis of ATP to ADP. The two transmembrane domains form a gated channel that is controlled by the state of the two intracellular NBDs.

**Figure 1:**
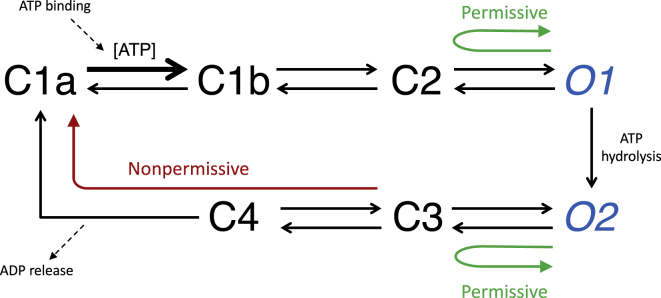
CFTR cycle model. Possible state transitions are indicated with black arrows. The C1a → C1b transition is sensitive to ATP concentration and is indicated with a bold arrow and is labeled [ATP]. States with open and closed ion channels are indicated in blue italics and black, respectively. Closings for which the reopening occurs in the same state are called permissive, depicted with green arrows; closings from O2 where the reopening is O1 are called nonpermissive, depicted with a red arrow. See also reference (4) and the supporting material.

Of key interest are permissive and nonpermissive closings of the CFTR ion channel, in the sense of permissive to rapid reopening the channel. Considering the kinetic model in Fig. 1 (see (4)), nonpermissive closings involve the release of ADP in order to enable the binding of ATP (5,6) and include the irreversible C4→C1a transition. On the other hand, permissive closings do not include this transition, meaning that they do not involve the release of ADP or ATP, leading to faster reopening. However, as these transitions occur on states in the same conductance level, the two types of closing cannot be directly distinguished by the patch clamp. The problem addressed by this report is to distinguish permissive and nonpermissive closings solely by performing signal processing on the patch-clamp trace*.*

Hidden-variable problems of this type have a long history in biophysics, e.g., (7). More recently, methods have been developed to automate idealization of noisy ion channel current recordings, including the expectation-maximization (EM) algorithm (8) and deep-learning (9) approaches. Nonparametric Bayesian approaches have been used to identify the number of kinetically distinct hidden states (10). Finally, many methods have been developed for estimation of the hidden state transition matrix, including maximum likelihood methods (11,12,13,14,15) and Bayesian approaches (16,17,18,19). Bayesian inference has also been used to estimate hidden kinetic states from synthetic patch-clamp measurements in (16), using a Markov chain Monte Carlo method.

In this report, we are interested in factor-graph-based inference algorithms, such as the sum-product algorithm (20,21), which can combine inference with EM-based estimation of unknown parameters (22,23). Algorithms of this type have been used in biomedical hidden-variable problems (24). Distinct from (16), the factor graph approach calculates posterior probabilities efficiently (and exactly if parameters are known). Elsewhere, applications of inference algorithms are found in diverse areas such as bioinformatics (25,26), biophysics (27,28), telecommunications, and, more recently, in machine learning (29).

The main contribution of this report is to show that permissive and nonpermissive closings of CFTR (i.e., the timing of ADP dissociation and ATP binding) can be accurately inferred from patch-clamp traces alone, without any additional equipment, model training, or prior knowledge about parameter values. In doing so, we apply a factor-graph-based inference algorithm; to our knowledge, our report is also the first to apply an inference algorithm of this kind to CFTR.

## Materials and methods

### Receptor model

We use a physical model of CFTR described in Fig. 1 (see (4)). In this seven-state model, states C1a, C1b, C2, C3, and C4 are fully closed, so we assume that ions are completely unable to pass through when CFTR is in these states, and thus, they have the same conductance level. States O1 and O2 are open states in which an ion current can flow through the channel. (The states are labeled so that the first letter indicates whether the channel is closed [C] or open [O].) Beginning with C1a, with a single ATP bound at the first ATP binding site, which is incapable of hydrolysis (30), the reversible transition to C1b occurs when a second ATP binds so that both binding sites are occupied. Because this step involves ATP binding, the rate depends on the concentration of ATP. The reversible transitions from C1b to C2 and from C2 to O1 are conformational changes resulting in an open CFTR, much like any ligand-gated channel such as the acetylcholine receptor (31). The transition from O1 to O2 is the first of two irreversible steps in the cycle, with one NBD-bound ATP undergoing hydrolysis to ADP. CFTR can then undergo reversible conformational changes from O2 to C3 and from C3 to C4, resulting in a closed pore. Finally, the second irreversible step occurs in the transition from C4 to C1a, where the NBD-bound ADP unbinds from CFTR, leaving one apo and one filled ATP binding site. This seven-state model is in agreement with the four-state simplified cyclic gating model of (32) and models distinguishing multiple closed and/or open states (33,34).

As noted in the introduction, we are interested in determining whether the channel closings are permissive or nonpermissive. Nonpermissive closings include the irreversible C4 to C1a transition, in which ADP unbinds and a binding site is available for ATP, while permissive closings do not. Thus, considering Fig. 1,

• A permissive closing has the same initial and final open states, i.e. O1→C2…C2→O1 or O2→C3…C3→O2, and
• A nonpermissive closing has different initial and final open states, i.e., O2→C3…C2→O1. From Fig. 1, the only way to do this is to proceed through the C4→C1a transition.

The states under the ellipsis (…) can be any valid sequence of closed states from Fig. 1, not necessarily the same state.

The kinetic microstates of CFTR can be modeled using a master equation of the form

(1) $$\frac{dP}{dt}=PR,$$

where P is a row vector with length equal to the number of kinetic microstates and R is a square matrix of kinetic rates for each possible state transition. In this formulation, $P_{i}$ is the probability that a receptor is in microstate i, while $R_{ij}$ is the transition rate from state i to state j. The rate matrix R and the full master equation are given in the supporting material.

### Patch-clamp signal model

Formally, our system contains a set V of observable conductance states, a set S of hidden kinetic microstates, and a mapping m:S→V from microstates to conductance states. For CFTR, we have

(2) V={0,1},

(3) S={C1a,C1b,C2,C3,C4,O1,O2},and

(4) $$m\left(s\right)=\left\{ \begin{array}{ll} 0, & s\in \{\text{C}1\text{a},\text{C}1\text{b},\text{C}2,\text{C}3,\text{C}4\}\\ 1, & s\in \{\text{O}1,\text{O}2\} \end{array}\right.\text{.}$$

The conductance states {0,1} correspond to the ion channel’s current when closed and open, respectively.

The patch clamp observes the channel current through additive noise and samples these observations with sampling time Δt to form discrete-time signals. Let $y=\left[y_{1},y_{2},\ldots ,y_{n}\right]\in R^{n}$ represent the sequence of observations for a single channel, and let $s=\left[s_{1},s_{2},\ldots ,s_{n}\right]\in S^{n}$ represent the corresponding microstates. Then, at the k th sample, the patch clamp measures

(5) $$y_{k}=I_{s_{k}}+n_{k},$$

where $I_{s_{k}}=Am\left(s_{k}\right)$ is the current flowing through the channel in state $s_{k}$, given by a constant A and the function m(s) in (4), and where $n_{k}$ forms a sequence of independent, identically distributed Gaussian random variables with zero mean and variance $\sigma^{2}$. Using the central limit theorem, it is reasonable to model the noise as Gaussian, particularly if a decimation filter is applied to the raw patch-clamp outputs (see the results section below).

The transitions of microstates $s_{k-1}\to s_{k}$ are modeled as a discrete-time Markov chain (35), again with a discrete time step Δt. The transition probability matrix for this Markov chain $Q=\left[Q_{ij}\right]=\left[\text{Pr}\left(s_{k}=j\;|\;s_{k-1}=i\right)\right]$ is given by the solution to (1):

(6) $$Q=e^{R\Delta t}.$$

### Inference, parameter estimation, and simulation

We use the sum-product algorithm (21) over the factor graph representing the sequence of states s to obtain the a posteriori distribution $p\left(s_{k}\;|\;y\right)$. Meanwhile, the transition probability matrix Q and noise variance $\sigma^{2}$ (and potentially the current amplitude A) are unknown a priori and must be estimated from the data. The EM algorithm (36) is a standard tool for this kind of simultaneous inference-estimation task. We employ a variant of the EM algorithm, known as the factor graph EM algorithm (22,37), which is intended for use alongside sum-product inference algorithms. The complete details of our algorithm are described in the supporting material.

Given $p\left(s_{k}\;|\;y\right)$, we define a confidence threshold C, 0≤C<1 and use the following decision rule to obtain the estimate ${\hat{s}}_{k}$ for each $s_{k}$:

(7) $${\hat{s}}_{k}=\left\{ \begin{array}{ll} \text{arg}\max_{s_{k}\in S}p\left(s_{k}\;|\;y\right), & p\left(s_{k}\;|\;y\right)>C,\\ \emptyset , & \text{otherwise}. \end{array}\right.$$

That is, $\hat{s}$ is the maximum a posteriori (MAP) estimate if the estimate exceeds C; otherwise, $\hat{s}$ is null (∅). Setting C=0 obtains the MAP estimate for all states $s_{k}$, while setting C>0 reduces the probability of false alarm.

We test our inference algorithm via Monte Carlo simulation by generating instances of discrete-time Markov chains with transition probability matrix Q (Eq. 6) and adding noise (Eq. 5). To evaluate the algorithm, we consider probability of false alarm $P_{\text{FA}}$ and probability of missed detection $P_{\text{MD}}$, also known, respectively, as type I and type II errors. First, using the ground-truth state sequence $s_{k}$, we make a list $L_{\text{GT}}$ of closings (i.e., any sequence OX→CX…CX→OX, with OX and CX representing any open or closed state, respectively, and where all states under the ellipsis (…) are closed states), and a list $L_{\text{GT}}^{\left(\text{np}\right)}$ of nonpermissive closings. Next, using the sequence of estimated states ${\hat{s}}_{k}$, we make similar lists of all estimated closings $L_{\text{E}}$ and estimated nonpermissive closings $L_{\text{E}}^{\left(\text{np}\right)}$. Represent the length of a list as, e.g., $\left|L_{\text{GT}}\right|$. Now, let $n_{\text{FA}}$ be the number of closings that appear in $L_{\text{E}}^{\left(\text{np}\right)}$ but not in $L_{\text{GT}}^{\left(\text{np}\right)}$ and let $n_{\text{MD}}$ be the number of closings that appear in $L_{\text{GT}}^{\left(\text{np}\right)}$ but not in $L_{\text{E}}^{\left(\text{np}\right)}$. Then,

(8) $$P_{\text{FA}}=\frac{n_{\text{FA}}}{\left|L_{\text{E}}^{\left(\text{np}\right)}\right|},\;P_{\text{MD}}=\frac{n_{\text{MD}}}{\left|L_{\text{GT}}^{\left(\text{np}\right)}\right|}.$$

### Patch-clamp measurements

Single CFTR channels were studied in inside-out patches pulled from *Xenopus* oocytes injected with cRNA encoding the wild-type channel, as previously described in (38). Briefly, to enable removal of the vitelline membrane, oocytes were placed in a bath solution containing (in mM) 200 monopotassium aspartate, 20 KCl, 1 MgCl_2_, 10 EGTA, and 10 HEPES (pH 7.2) adjusted with KOH. Gigaohm seals were formed with patch pipettes pulled from borosilicate glass and filled with solution containing (in mM) 150 N-methyl-D-glutamine chloride, 5 MgCl_2_, and 10 TES buffer (pH 7.5). After excision of the patch, CFTR channels were activated by bath solution containing 150 mM N-methyl-D-glutamine chloride, 1.1 mM MgCl_2_, 2 mM Tris-EGTA, 10 mM TES buffer, 1 mM MgATP, and 127 U/ml PKA (pH 7.5). Currents were recorded at V_M_ = −100 mV using an Axopatch 200B amplifier, with filtering at 0.1–1 kHz. The sampling rate of the patch clamp data was 2 kHz.

### Code

Code and raw patch-clamp traces used to generate all results in this report are publicly available on Zenodo (see (39)). We do not include simulated patch-clamp traces in this dataset, but simulations can be generated using the code we provide. The code repository also includes a Jupyter notebook and raw data for generating all results in this report.

## Results

### Analysis using simulated patch-clamp measurements

Here, we give results obtained from Monte Carlo simulations of the CFTR ion channel. To generate simulated patch-clamp results, we use example model parameters for the seven-state CFTR model given in Table 1. These example values are not provided to the inference algorithm, so performance does not in general depend on their accuracy.

**Table 1:** Parameters for the CFTR channel, corresponding to the model in Figure 1

Origin state	Destination state						
	C1a	C1b	C2	O1	O2	C3	C4
C1a		$9.0\cdot 10^{3}$ (M s)^−1^ [ATP]					
C1b	5.0 s^-1^		7.7 s^−1^				
C2		5.8 s^−1^		4.9 s^−1^			
O1			10.0 s^−1^		7.1 s^−1^		
O2						3.0 s^−1^	
C3					7.0 s^−1^		6.0 s^−1^
C4	1.7 s^−1^					12.8 s^−1^	

Parameters are derived to match the wild-type (high $P_{o}$) model from (4). A blank entry indicates that the transition is impossible. [ATP] indicates molar concentration of ATP.

#### Properties of nonpermissive closings

**Figure 2:**
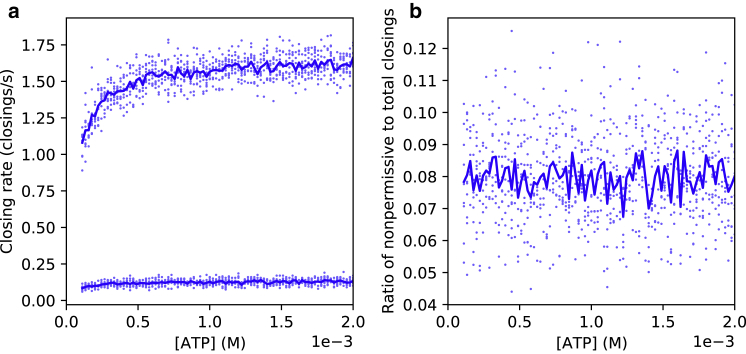
Prevalence of nonpermissive closings. (*a*) Rate of all channel closings (*top line*) and nonpermissive closings (*bottom line*) versus ATP concentration. (*b*) Ratio of nonpermissive to total closings versus ATP concentration. Dots represent the outcomes of each simulation run, and lines represent mean value at each concentration. Sampling rate = 100 Hz; rate parameters from Table 1.

In Fig. 2, we used our simulator to generate ground-truth state sequences $s_{k}$; we then count the number of nonpermissive closings $\left|L_{\text{GT}}^{\left(\text{np}\right)}\right|$ and the total number of closings $\left|L_{\text{GT}}\right|$ and divide by time to get rates. In the figure, we see that nonpermissive closings occur at a much lower rate than permissive closings. While the rate of closings depends on ATP concentration due to the cyclical nature of CFTR gating, the ratio of nonpermissive to total closings remains nearly independent of concentration. These observations are consistent with the dynamics of our model: a low ATP concentration will lead to a longer dwell time in state C1a, thus increasing the interval between openings without affecting the rates of transitions at the boundary between open and closed. Moreover, nonpermissive closings are relatively rare, with a ratio of roughly 1 nonpermissive closing per 12 total closings.

#### Missed detection and false alarm probability

**Figure 3:**
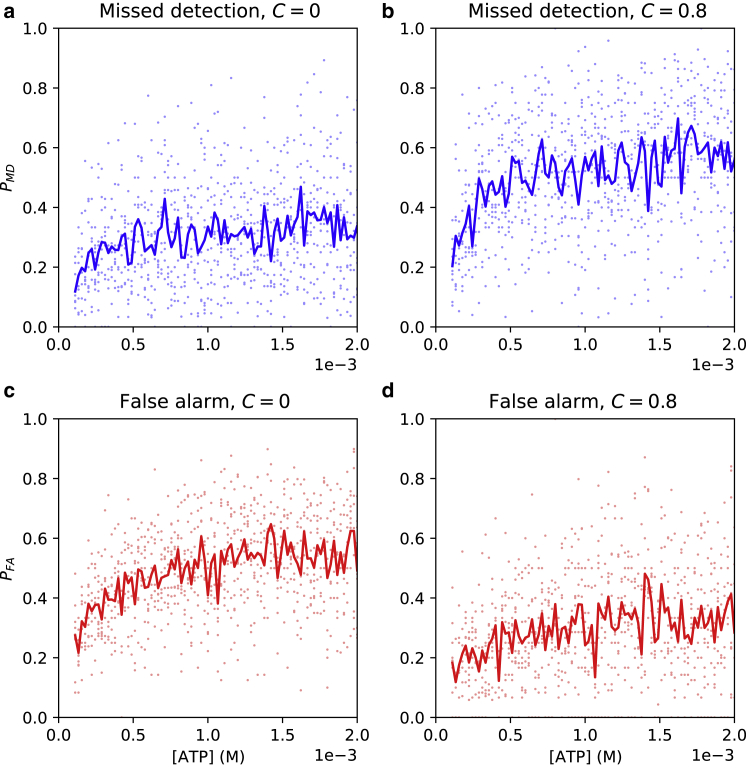
Missed detection and false alarm probabilities. (*a* and *b*) Missed detection probability versus ATP concentration for confidence thresholds C=0 and C=0.8, respectively. (*c* and *d*) False alarm probability versus ATP concentration for *C* = 0 and C=0.8, respectively. Dots represent each simulation run, while lines represent the average at each concentration. Sampling rate = 100 Hz, 20,000 samples, 400 EM iterations, $\sigma^{2}=0.02$; rate parameters from Table 1.

In Fig. 3, we give missed detection and false alarm probabilities, $P_{\text{MD}}$ and $P_{\text{FA}}$, using our algorithm (see Eq. 8). Setting the confidence threshold C=0 gives the MAP estimate of each state (see Eq. 7); we see a low $P_{\text{MD}}$, meaning few nonpermissive closings are missed, but $P_{\text{FA}}$ is relatively high, i.e., around 50%. (Since nonpermissive closings are rare, cf. Fig. 2 *b*; this is still far better than random guessing.) With a higher confidence threshold of C=0.8, $P_{\text{FA}}$ is reduced at the expense of increased $P_{\text{MD}}$. This is explained by noting that lower-confidence state estimates are discarded, so those closings will be missed by the algorithm. This demonstrates that C can be adjusted to trade off $P_{\text{FA}}$ against $P_{\text{MD}}$. The performance of the algorithm is dependent on ATP concentration, with error rates increasing as concentration increases.

#### Application to CFTR mutants

We modified the rate matrix in Table 1 to represent the K1250A CFTR mutant (see (4)). Complete details and results, similar to Figs. 2 and 3, are provided in the supporting material.

### Application to experimentally obtained patch-clamp measurements

**Figure 4:**
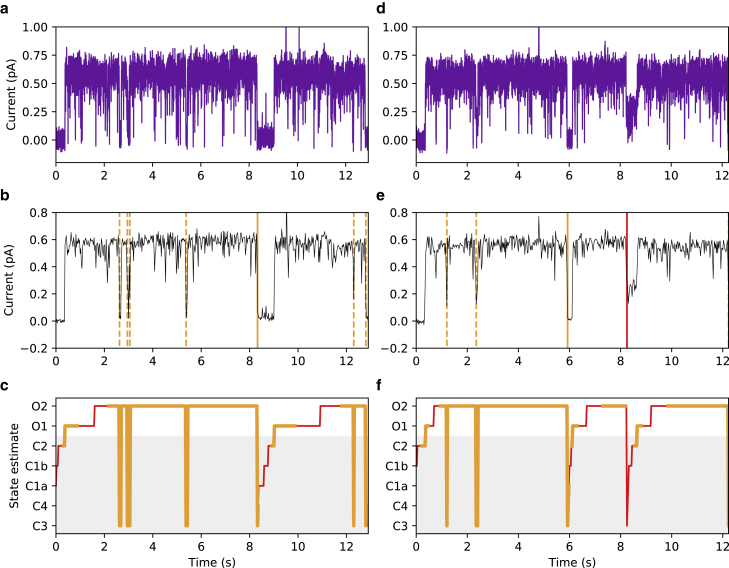
CFTR patch-clamp measurements (raw and preprocessed) along with the corresponding inferred states for two different experiments, one in each column. (*a* and *d*, *top row*) Measured patch-clamp current. (*b* and *e*, *middle row*) Patch-clamp current signal after decimation; this signal is provided to the inference algorithm. Detected closings are depicted on these figures, with dashed/solid vertical lines, respectively, indicating permissive/nonpermissive closings; orange/red lines represent detected closings that, respectively, exceed/do not exceed C=0.8. (*c* and *f*, *bottom row*) Inferred state after 400 EM iterations; orange/red lines represent state estimates that, respectively, exceed/do not exceed C=0.8.

In Fig. 4, we show the application of our algorithm to lab-obtained CFTR patch-clamp measurements. We show two examples corresponding to two different experiments. For techniques used to obtain these measurements, see the materials and methods section.

As the patch-clamp signal is oversampled compared with the kinetics of CFTR, we decimate the signal by a factor of 50, applying a block-averaging decimation filter (taking the sample average over nonoverlapping blocks of 50 samples). The decimation step is performed to reduce the noise at high frequencies, which contains very little useful information about the signal, while preserving the features of interest at lower frequencies, improving the performance of the algorithm. We show the decimated signal in the middle plots of Fig. 4, overlaid with vertical lines indicating the closings found by our algorithm, both permissive and nonpermissive. In the bottom plots of Fig. 4, we give the state estimates ${\hat{s}}_{k}$ found by the algorithm, with different colors indicating whether or not the initial and final estimated states both exceed a confidence threshold of C=0.8. From the raw data and preprocessed traces, abrupt transitions from high to low current correspond well with the detected closings.

## Discussion

We show that an algorithmic tool reveals the precise microstate kinetics of CFTR. By revealing permissive and nonpermissive closings, we can precisely estimate the timing of each nucleotide unbinding event, a key step in CFTR’s kinetic model. Furthermore, our method may be used to study the effect of reagents that are known to affect hidden-state kinetics of CFTR, such as scorpion venom (4); future experiments in this direction might also be used to analyze pharmaceuticals that target CFTR. More generally, beyond permissive and nonpermissive closings, this method gives the designer of an experiment a novel and fine-grained algorithmic tool to discover changes to the behavior of receptor proteins. For example, this method could be used to determine the fine-grained, microstate-by-microstate effects of particular agonists or mutations, in CFTR or other receptors.

## Author contributions

A.S.M., N.A.M., and A.W.E. wrote the article. A.W.E. designed the research, wrote the simulation code, and carried out the simulations. N.A.M. and G.C. performed the patch clamp experiments. P.J.T., W.D.H., and R.S.W. edited the article and contributed research ideas.

## Supporting material

Document S1. Supporting material and Figures S1–S4

Document S2. Article plus supporting material
