# Supplementary material for: Permissive and nonpermissive channel closings in CFTR revealed by a factor graph inference algorithm
Source: Biophys Rep (N Y). 2022 Oct 19;2(4):100083. doi: 10.1016/j.bpr.2022.100083 (PMC9680790; doi:10.1016/j.bpr.2022.100083)
Supplement: Document S1. Supporting material and Figures S1–S4 [file mmc1.pdf]

Biophysical Reports, Volume *2*

# Supplemental information

# Permissive and nonpermissive channel closings in CFTR revealed by a factor graph inference algorithm

**Alexander S. Moffett, Guiying Cui, Peter J. Thomas, William D. Hunt, Nael A. McCarty, Ryan S. Westafer, and Andrew W. Eckford**

# Supplemental Information

Equation references of the form (1) refer to equations in the main paper, while references of the form (S1) refer to equations in this supplemental information.

# 1 Rate matrix

The transition rates are given in the rate matrix (with rows and columns, in order, corresponding to C1a, C1b, C2, O1, O2, C3, and C4):

$$R = \begin{bmatrix} R_{11} & k_{\mathrm{C1a}\to\mathrm{C1b}} & 0 & 0 & 0 & 0 & 0 \\ k_{\mathrm{C1b}\to\mathrm{C1a}} & R_{22} & k_{\mathrm{C1b}\to\mathrm{C2}} & 0 & 0 & 0 & 0 \\ 0 & k_{\mathrm{C2}\to\mathrm{C1b}} & R_{33} & k_{\mathrm{C2}\to\mathrm{O1}} & 0 & 0 & 0 \\ 0 & 0 & k_{\mathrm{O1}\to\mathrm{C2}} & R_{44} & k_{\mathrm{O1}\to\mathrm{O2}} & 0 & 0 \\ 0 & 0 & 0 & 0 & R_{55} & k_{\mathrm{O2}\to\mathrm{C3}} & 0 \\ 0 & 0 & 0 & 0 & k_{\mathrm{C3}\to\mathrm{O2}} & R_{66} & k_{\mathrm{C3}\to\mathrm{C4}} \\ k_{\mathrm{C4}\to\mathrm{C1a}} & 0 & 0 & 0 & 0 & k_{\mathrm{C4}\to\mathrm{C3}} & R_{77} \end{bmatrix} \tag{S1}$$

with the diagonal entries $R_{ii}$ set so that each row of $R$ sums to zero, and with $R_{ij} = 0$ indicating that the transition $i \to j$ is forbidden. Table 1 gives example values for each rate, noting that the value of $k_{\mathrm{C1a}\to\mathrm{C1b}}$ is dependent on the environmental concentration of ATP.

# 2 Master equation

Consider the master equation from equation (1) in the main paper. Here $P$ gives the occupancy probabilities for each microstate. In the case of the CFTR receptor,

$$P = \Big[P(\mathrm{C1a}), P(\mathrm{C1b}), P(\mathrm{C2}), P(\mathrm{O1}), P(\mathrm{O2}), P(\mathrm{C3}), P(\mathrm{C4})\Big]. \tag{S2}$$

Using (S2) and (S1), the continuous-time master equation is fully expanded by

$$\frac{dP(\mathrm{C1a})}{dt} = -k_{\mathrm{C1a}\to\mathrm{C1b}}P(\mathrm{C1a}) + k_{\mathrm{C4}\to\mathrm{C1a}}P(\mathrm{C4}) + k_{\mathrm{C1b}\to\mathrm{C1a}}P(\mathrm{C1b}) \tag{S3}$$

$$\frac{dP(\mathrm{C1b})}{dt} = -(k_{\mathrm{C1b}\to\mathrm{C2}} + k_{\mathrm{C1b}\to\mathrm{C1a}})P(\mathrm{C1b}) + k_{\mathrm{C1a}\to\mathrm{C1b}}P(\mathrm{C1a}) + k_{\mathrm{C2}\to\mathrm{C1b}}P(\mathrm{C2}) \tag{S4}$$

$$\frac{dP(\mathrm{C2})}{dt} = -(k_{\mathrm{C2}\to\mathrm{O1}} + k_{\mathrm{C2}\to\mathrm{C1b}})P(\mathrm{C2}) + k_{\mathrm{C1b}\to\mathrm{C2}}P(\mathrm{C1a}) + k_{\mathrm{O1}\to\mathrm{C2}}P(\mathrm{O1}) \tag{S5}$$

$$\frac{dP(\mathrm{O1})}{dt} = -(k_{\mathrm{O1}\to\mathrm{O2}} + k_{\mathrm{O1}\to\mathrm{C2}})P(\mathrm{O1}) + k_{\mathrm{C2}\to\mathrm{O1}}P(\mathrm{C2}) \tag{S6}$$

$$\frac{dP(\mathrm{O2})}{dt} = -k_{\mathrm{O2}\to\mathrm{C3}}P(\mathrm{O2}) + k_{\mathrm{O1}\to\mathrm{O2}}P(\mathrm{O1}) + k_{\mathrm{C3}\to\mathrm{O2}}P(\mathrm{C3}) \tag{S7}$$

$$\frac{dP(\mathrm{C3})}{dt} = -(k_{\mathrm{C3}\to\mathrm{C4}} + k_{\mathrm{C3}\to\mathrm{O2}})P(\mathrm{C3}) + k_{\mathrm{O2}\to\mathrm{C3}}P(\mathrm{O2}) + k_{\mathrm{C4}\to\mathrm{C3}}P(\mathrm{C4}) \tag{S8}$$

$$\frac{dP(\mathrm{C4})}{dt} = -(k_{\mathrm{C4}\to\mathrm{C1a}} + k_{\mathrm{C4}\to\mathrm{C3}})P(\mathrm{C4}) + k_{\mathrm{C3}\to\mathrm{C4}}P(\mathrm{C3}). \tag{S9}$$

Example values of each rate are given in Table 1 in the main paper. We suppress the dependence of $k_{\mathrm{C1a}\to\mathrm{C1b}}$ on ATP concentration in our notation for the sake of compactness, but as noted in Table 1 the effects of ATP concentration are fully considered in the model.

# 3 Factor graphs and the sum-product algorithm

Factor graph inference is a method of probabilistic analysis. Let $s$ be a random variable, which is of interest but cannot be directly observed, such as the kinetic microstates of CFTR. Let $y$ be a collection of random variables that are observed, and correlated with $s$ through a conditional probability $p(y \mid s)$; for example, $y$ could be a collection of partial and/or noisy observations of $s$, such as the CFTR patch clamp measurements in this paper. Then the goal of factor graph inference is to calculate the conditional probability $p(s \mid y)$ of $s$ given all knowledge $y$ using Bayes' rule, i.e.,

$$p(s \mid y) = \frac{p(y \mid s)p(s)}{\sum_s p(y \mid s)p(s)}. \tag{S10}$$

The quantity $p(s \mid y)$ is known as the *a posteriori* probability, as it is calculated "after" observing $y$, and includes all available evidence about $s$. (This is as opposed to $p(s)$, called the *a priori* probability, which is our belief about $s$ "before" observing any evidence $y$.) If we want to guess the value of $s$, then picking the value that maximizes $p(s|y)$, i.e. $\arg\max_s p(s|y)$, is known to be the decision rule with the smallest probability of error, and is called the maximum *a posteriori* (MAP) estimate.

The simplicity of (S10) belies the high computational complexity of this operation, particularly for large probabilistic models. Thus, efficient algorithms such as the sum-product algorithm are highly desirable. For more details and generalizations, the reader is encouraged to consult [1].

In CFTR, we observe noisy patch clamp currents $y = [y_1, y_2, \ldots, y_n]$, and we want to infer the underlying kinetic states $s = [s_1, s_2, \ldots, s_n]$. The sum-product algorithm for this problem is derived as follows. The probability mass function of the state sequence $s$ can be written

$$p(s) = \prod_{k=1}^{n} p(s_k \mid s_{k-1}), \tag{S11}$$

where $s_0$ is null, i.e., $p(s_1 \mid s_0) = p(s_1)$. Including the noisy current observations $y$ in the model, we can first write

$$p(y_k \mid s_k) = \frac{1}{\sqrt{2\pi\sigma^2}} \exp\left(-\frac{(y_k - I_{s_k})^2}{2\sigma^2}\right). \tag{S12}$$

where $I_{s_k}$ is the current through the channel in state $s_k$. Finally, we have the joint probability mass function

$$p(y, s) = \prod_{k=1}^{n} p(y_k \mid s_k) p(s_k \mid s_{k-1}). \tag{S13}$$

The stochastic model $p(y, s)$ can be represented on a *factor graph*, where nodes representing variables $s_1, \ldots, s_n$ and $y_1, \ldots, y_n$ are connected to nodes representing factors in (S13), and an edge is drawn from variable to factor if the factor is a function of the variable. The factor graph for (S13) is depicted in Figure S1.

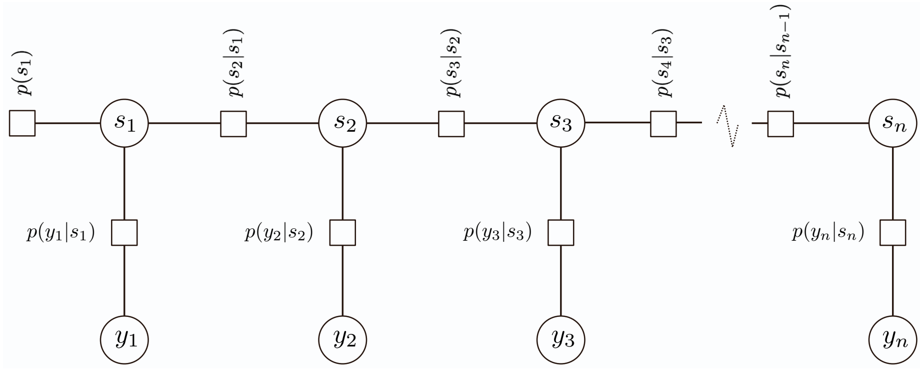


Figure S1: Factor graph for the patch clamp observations.

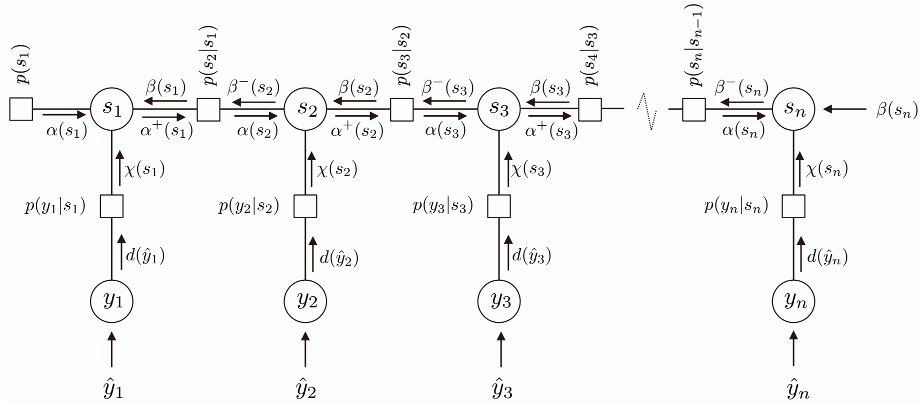


Figure S2: Factor graph for the patch clamp observations with sum-product messages indicated on each edge (cf. Figure S1).

We are interested in the *a posteriori* probability $p(s_k \mid y)$, i.e., the distribution over $s_k$ knowing the observed (noisy) current for all time. This can be calculated using the forward-backward algorithm, a special case of the sum-product algorithm, which is a message-passing algorithm over the factor graph.

The flow of messages within the factor graph is depicted in Figure S2. *Generally speaking, messages on the outgoing edges from a node are calculated using all incoming messages to that node,* ***except*** *the incoming message along the same outgoing edge.*

In terms of the variable nodes $s_k$, the forward message $\alpha_v^+(s_k)$ is the component-wise product of the incoming forward message $\alpha_v(s_k)$ and the channel message $\chi(s_k)$ (defined below); the backward message $\beta_v^-(s_k)$ is the component-wise product of the incoming backward message $\beta_v(s_k)$ and the channel message $\chi(s_k)$ (defined below). It will be convenient to express the forward messages $\alpha$ as row vectors, and the backward messages $\beta$ as column vectors. Representing these message calculations as matrix multiplications,

$$\alpha_v^+(s_k) = \alpha_v(s_k)\text{diag}\Big(\chi(s_k)\Big) \tag{S14}$$

$$\beta_v^-(s_k) = \text{diag}\Big(\chi(s_k)\Big)\beta_v(s_k) \tag{S15}$$

In terms of the factor nodes $p(y_k \mid s_k)$, $p(s_k \mid s_{k-1})$, each factor node "contains" the factor, and this factor is incorporated into the message calculation. As the factors are conditional probability functions in two variables, it will be convenient to represent the factors as matrices, with the conditional variable remaining constant along the rows, and the probabilistic variable remaining constant along the columns; this implies the matrices are row-stochastic. Message calculations are performed as follows:

- At $p(y_k \mid s_k)$, the channel message $\chi(s_k)$ is the vector

$$\chi(s_k) = \Big[\chi(s_k = 1), \chi(s_k = 2), \ldots, \chi(s_k = |\mathcal{S}|)\Big] \tag{S16}$$

  with slight abuse of the notation: $s_k = i$ means $s_k$ is the $i$th element of $\mathcal{S}$

from (3). Moreover,

$$\chi(s_k = i) = p(y_k \mid s_k = i), \tag{S17}$$

where $p(y_k \mid s_k = i)$ is given by (S12). (For clarity, to obtain this value we substitute the patch clamp current observation $y_k$ into (S17); with $y_k$ and $s_k = i$ both defined, the result is a scalar constant.)

- At $p(s_k \mid s_{k-1})$, we have an outgoing forward message $\alpha(s_k)$ and an outgoing backward message $\beta(s_{k-1})$, with an incoming forward message $\alpha^+(s_{k-1})$ and incoming backward message $\beta^-(s_k)$. The internal factor $p(s_k \mid s_{k-1})$ is given by the transition probability matrix $Q$. The message calculation is given by

$$\alpha(s_k) = \alpha^+(s_{k-1})Q \tag{S18}$$

$$\beta(s_{k-1}) = Q\beta^-(s_k) \tag{S19}$$

Combining (S14) and (S18), and (S15) and (S19), we can write

$$\alpha(s_k) = \alpha(s_{k-1})\mathrm{diag}\Big(\chi(s_{k-1})\Big)Q \tag{S20}$$

$$\beta(s_k) = Q\mathrm{diag}\Big(\chi(s_{k+1})\Big)\beta(s_{k+1}) \tag{S21}$$

Next we must specify the order in which operations occur, i.e., the message-passing schedule:

- The channel messages $\chi(s_k)$ are precalculated.
- For the forward messages, the initial forward message $\alpha(s_1)$ is the steady-state distribution associated with $Q$. We then iteratively calculate (S20) for each $k = 2, 3, \ldots$.
- For the backward messages, we set the initial backward message $\beta(s_n) = [1, 1, \ldots, 1]$ (this message is uninformative, as the future gives us no information). We then iteratively calculate (S21) for each $k = n-1, n-2, \ldots$.

Finally, to calculate the *a posteriori* probability $p(s_k \mid y)$, we perform the complete message-passing schedule described above. Following the message calculations, we take the product of all messages inbound to variable node $s_k$, and normalize (to force the product to sum to 1). Letting $\odot$ represent componentwise multiplication:

$$p(s_k \mid y) = \frac{\alpha(s_k) \odot \beta(s_k) \odot \chi(s_k)}{\sum_{s_k} \alpha(s_k) \odot \beta(s_k) \odot \chi(s_k)}. \tag{S22}$$

(Normalizations can also be performed at any intermediate stage of the message calculations, and this is often done for numerical stability.)

# 4 Factor Graph EM algorithm

The message-passing algorithm given in Section 3 requires knowledge of the system parameters, i.e., the state transition probability matrix $Q$, the noise variance $\sigma^2$, and the current values $I_{s_k}$. Here we derive a factor graph EM algorithm to obtain these estimates; this algorithm is intended for use alongside the sum-product family of inference algorithms.

## 4.1 Estimating $Q$

First consider the estimation of $Q$. The factor graph EM algorithm proceeds iteratively in two steps. In the E-step, we calculate a function $\Phi(Q; \bar{Q})$ of both the previous estimate $\bar{Q}$ and the actual parameter value $Q$; then in the M-step, we maximize $\Phi(Q; \bar{Q})$ with respect to $Q$. The maximizing value becomes the value of $\bar{Q}$ in the next E-step, and so on until the desired number of iterations is reached. One E-step and one M-step constitutes one iteration of the EM algorithm.

With observations $y$, hidden states $s$, matrix $Q$ of parameters (i.e. the state transition probability matrix), and estimate $\bar{Q}$ of the parameters, we have

$$\Phi(Q; \bar{Q}) = E_{\bar{Q}}\Big[\log p(y, s; Q) \mid y\Big] \tag{S23}$$

where the subscript $\bar{Q}$ indicates that the expectation is taken while setting $Q = \bar{Q}$. From (S13) we can write

$$\begin{aligned}
&E_{\bar{Q}}\Big[\log p(y, s; Q) \mid y\Big] \\
&= E_{\bar{Q}}\left[\log \prod_{k=1}^{n} p(y_k \mid s_k) p(s_k \mid s_{k-1}; Q) \mid y\right] && \text{(S24)} \\
&= \sum_{k=1}^{n} E_{\bar{Q}}\Big[\log p(y_k \mid s_k) \mid y\Big] + \sum_{k=1}^{n} E_{\bar{Q}}\Big[\log p(s_k \mid s_{k-1}; Q) \mid y\Big]. && \text{(S25)}
\end{aligned}$$

The first term in (S25) is constant with respect to $Q$ and is therefore not important for the rest of the derivation, so we will absorb it into a constant $C$. Now we have

$$\begin{aligned}
&E_{\bar{Q}}\Big[\log p(y, s; Q) \mid y\Big] \\
&= \sum_{k=1}^{n} E_{\bar{Q}}\Big[\log p(s_k \mid s_{k-1}; Q) \mid y\Big] + C && \text{(S26)} \\
&= \sum_{k=1}^{n} \sum_{s_k, s_{k-1}} p(s_k, s_{k-1} \mid y; \bar{Q}) \log p(s_k \mid s_{k-1}; Q) + C. && \text{(S27)}
\end{aligned}$$

The term $p(s_k, s_{k-1} \mid y; \bar{Q})$ can be obtained directly from the sum-product algorithm, as the (normalized) product of all messages incident to the factor node $p(s_k \mid s_{k-1})$, setting $Q = \bar{Q}$ throughout the factor graph. That is, forming a $|\mathcal{S}| \times |\mathcal{S}|$ matrix $M^{(k)} = [M_{ij}^{(k)}]$, where

$$M^{(k)} = \text{diag}(\alpha^{+}(s_{k-1}))\bar{Q}\text{diag}(\beta^{-}(s_k)), \tag{S28}$$

we have that $M_{ij}^{(k)} = p(s_k = j, s_{k-1} = i \mid y; \bar{Q})$, i.e., we can read the values of $p(s_k, s_{k-1} \mid y; \bar{Q})$ from $M^{(k)}$. Finally, returning to (S27),

$$\begin{aligned}
&E_{\bar{Q}}\Big[\log p(y, s; Q) \mid y\Big] \\
&= \sum_{k=1}^{n} \sum_{s_k, s_{k-1} = (j,i)} M_{ij}^{(k)} \log Q_{ij} + C. && \text{(S29)}
\end{aligned}$$

Calculation of (S29) constitutes the E-step of the EM algorithm.

The M-step is performed as follows. Recall that $Q$ is row-stochastic, so for constant $i$, $Q_{ij}$ forms a probability mass function in $j$. Now (S29) can be

rewritten

$$\begin{aligned} & E_{\bar{Q}}\Big[\log p(y, s; Q) \mid y\Big] \\ & = \sum_{s_{k-1}} \sum_{s_k} \sum_{k=1}^{n} M_{ij}^{(k)} \log Q_{ij} + C && \text{(S30)} \\ & = \sum_{s_{k-1}} Z_i \sum_{s_k} \frac{M_{ij}}{Z_i} \log Q_{ij}, && \text{(S31)} \end{aligned}$$

where

$$M_{ij} = \sum_{k=1}^{n} M_{ij}^{(k)} \tag{S32}$$

$$Z_i = \sum_{s_k} M_{ij} \tag{S33}$$

Each inner sum of the form $\sum_{s_k} \frac{M_{ij}}{Z_i} \log Q_{ij}$ is maximized by setting

$$\bar{Q}^{+}_{ij} = \frac{M_{ij}}{Z_i}. \tag{S34}$$

Thus, the M-step is accomplished by forming the matrix $\bar{Q}^{+} = [\bar{Q}^{+}_{ij}]$, with $\bar{Q}^{+}_{ij}$ given by (S34). Finally, in the next iteration, $\bar{Q}^{+}$ is fed back to the E-step as $\bar{Q}$; if no more iterations are desired, then $\bar{Q}^{+}$ is the final estimate.

## 4.2 Estimating $\sigma^2$

Here we derive the estimation of $\sigma^2$, which occurs simultaneously with the estimation of $Q$. Modifying (S25), we can write

$$\begin{aligned} & E_{\bar{Q},\bar{\sigma}^2}\Big[\log p(y, s; Q, \sigma^2)\Big] \\ & = \sum_{k=1}^{n} E_{\bar{Q},\bar{\sigma}^2}\Big[\log p(y_k \mid s_k; \sigma^2) \mid y\Big] + \sum_{k=1}^{n} E_{\bar{Q},\bar{\sigma}^2}\Big[\log p(s_k \mid s_{k-1}; Q) \mid y\Big]. && \text{(S35)} \end{aligned}$$

In (S35), note that only the first term is a function of $\sigma^2$, and only the second term is a function of $Q$. Thus, the E- and M-steps with respect to each parameter can be performed independently. (For $Q$ these steps are described in detail in the previous section.)

Estimation of $\sigma^2$ for additive Gaussian noise models such as (5) is a frequently-used application of the EM algorithm, but we give the derivation here for completeness. Starting with the first term in (S35), we can write

$$\sum_{k=1}^{n} E_{\bar{Q},\bar{\sigma}^2}\Big[\log p(y_k \mid s_k; \sigma^2) \mid y\Big] = \sum_{k=1}^{n} \sum_{s_k} p(s_k \mid y; \bar{Q}, \bar{\sigma}^2) \log p(y_k \mid s_k; \sigma^2) \tag{S36}$$

The term $p(s_k \mid y; \bar{Q}, \bar{\sigma}^2)$ is obtained from the sum-product algorithm, and is the *a posteriori* probability of $s_k$ given $y$, obtained from (S22). Meanwhile, letting $I_{s_k}$ represent the current flowing through the patch clamp in each state $s_k$, from (5) we have

$$\log p(y_k \mid s_k; \sigma^2) = -\frac{1}{2} \log 2\pi\sigma^2 - \frac{(y_k - I_{s_k})^2}{2\sigma^2}. \tag{S37}$$

Let

$$W = \frac{1}{n} \sum_{k=1}^{n} \sum_{s_k} (y_k - I_{s_k})^2 p(s_k \mid y; \bar{Q}, \bar{\sigma}^2). \tag{S38}$$

Then (S36) becomes

$$E_{\bar{Q},\bar{\sigma}^2}\Big[\log p(y_k \mid s_k; \sigma^2) \mid y\Big] = -n\frac{1}{2} \log 2\pi\sigma^2 - n\frac{W}{2\sigma^2}. \tag{S39}$$

Calculation of (S39) and (S29) constitute the E-step of this EM algorithm.

In the M-step, it can be shown that the maximizing value of $\sigma^2$ in (S39) is

$$\sigma^2 = W. \tag{S40}$$

Setting $\sigma^2$ as in (S40) and $Q_{ij}$ as in (S34) completes the M-step of this EM algorithm.

# 5 Estimating $I_{s_k}$

Physical patch clamp measurements might not be calibrated, in which case $I_{s_k}$ is an unknown parameter of the system. In this case, $I_{s_k}$ can also be estimated by the EM algorithm: let $I_{\mathcal{O}}$ and $I_{\mathcal{C}}$ represent the new parameters, i.e. the current while the channel is open and closed, respectively.

In this algorithm, starting with (S37), the E-step is still given by (S39), but $W$ is a function of the parameters $I_O$ and $I_C$. In the M-step, we maximize (S37) with respect to $I_{\mathcal{O}}$ and $I_{\mathcal{C}}$. It can be shown that

$$\bar{I}_{\mathcal{O}} = \frac{\sum_{k=1}^{n} y_k \Pr(s_k \in \mathcal{O})}{\sum_{k=1}^{n} \Pr(s_k \in \mathcal{O})} \tag{S41}$$

$$\bar{I}_{\mathcal{C}} = \frac{\sum_{k=1}^{n} y_k \Pr(s_k \in \mathcal{C})}{\sum_{k=1}^{n} \Pr(s_k \in \mathcal{C})} \tag{S42}$$

maximizes (S39) for $I_{\mathcal{O}}$ and $I_{\mathcal{C}}$, where $\mathcal{O}$ is the set of open states, and $\Pr(s_k \in \mathcal{O}) = \sum_{s_k \in \mathcal{O}} p(s_k \mid y)$ is the posterior probability that $s_k$ is in the open state (similarly for $\Pr(s_k \in \mathcal{C})$, where $\mathcal{C}$ is the set of closed states, replacing $\mathcal{O}$ with $\mathcal{C}$ everywhere).

For convenience, we employ an M-step that maximizes with respect to $I_{s_k}$ first (keeping the previous estimate of $\sigma^2$), and subsequently maximizes with respect $\sigma^2$. That is, in (S38), when obtaining $\bar{\sigma}^2$ we first obtain $\bar{I}_{\mathcal{O}}$ and $\bar{I}_{\mathcal{C}}$ using the above equations, and then fix $I_{s_k}$ at the appropriate value when calculating $W$. This is strictly called a *generalized EM algorithm* (GEM) and retains the EM algorithm's property of monotonically increasing likelihood [2].

# 6 Results for K1250A mutant

In Figures S3 and S4, we reproduce Figures 2 and 3 (respectively) from the main paper, but using the K1250A mutant of CFTR. We use the same rate matrix $R$ as in Table 1 of the main paper, with two modifications: $R_{\mathrm{C2}\to\mathrm{O1}} = 0.2$ and $R_{\mathrm{O2}\to\mathrm{C3}} = 0.1$ (changed from original values of 4.9 and 3.0, respectively). These parameter changes reflect physiological and statistical properties of K1250A observed in [3]. Compared with the wild type, we observe that the closing rate is an order of magnitude smaller and that the ratio of nonpermissive to total closings is approximately the same, as expected. The detection performance is similar in terms of probability of false alarm, but significantly improved in terms of probability of missed detection. However, given the rarity of closings, the variance observed in these simulations is relatively high.

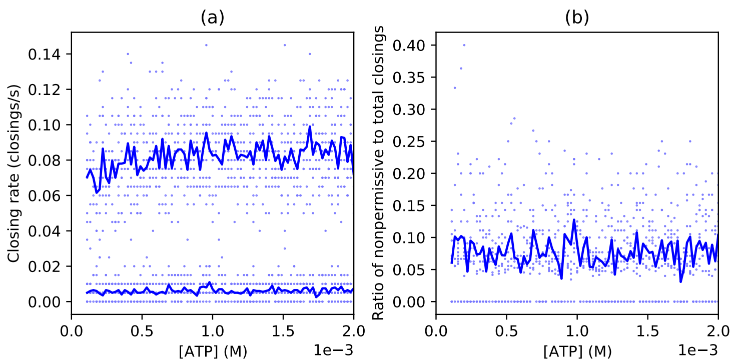


Figure S3: Prevalence of nonpermissive closings for the K1250A mutant. *Subfigure (a)*: Rate of all channel closings (top line) and nonpermissive closings (bottom line) versus ATP concentration. *Subfigure (b):* Ratio of nonpermissive to total closings versus ATP concentration. Dots represent the outcomes of each simulation run, lines represent mean value at each concentration. Sampling rate = 100 Hz, rate parameters as described above.

# References

[1] Frank R Kschischang, Brendan J Frey, and H-A Loeliger. Factor graphs and the sum-product algorithm. *IEEE Trans. Inf. Theory*, 47(2):498–519, 2001.

[2] Arthur P Dempster, Nan M Laird, and Donald B Rubin. Maximum likelihood from incomplete data via the EM algorithm. *J. R. Stat. Soc. Series B Stat. Methodol.*, 39(1):1–22, 1977.

[3] Matthew D Fuller, Zhi-Ren Zhang, Guiying Cui, and Nael A McCarty. The block of CFTR by scorpion venom is state-dependent. *Biophys. J.*, 89(6):3960–3975, 2005.

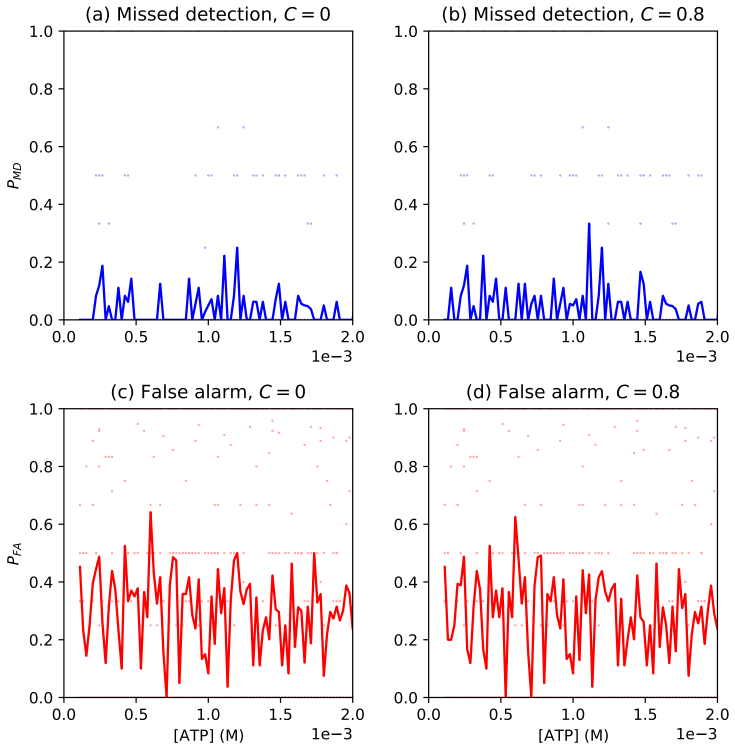


Figure S4: Missed detection and false alarm probabilities for the K1250A mutant. *Subfigures (a), (b):* Missed detection probability versus ATP concentration for confidence threshold $C = 0$ and $C = 0.8$, respectively. *Subfigures (c), (d):* False alarm probability versus ATP concentration for $C = 0$ and $C = 0.8$, respectively. Dots represent each simulation run, while lines represent the average at each concentration. Sampling rate = 100 Hz, 20000 samples, 400 EM iterations, $\sigma^2 = 0.02$, rate parameters as described above.
