# Supplementary material for: Permissive and nonpermissive channel closings in CFTR revealed by a factor graph inference algorithm
Source: Biophys Rep (N Y). 2022 Oct 19;2(4):100083. doi: 10.1016/j.bpr.2022.100083 (PMC9680790; doi:10.1016/j.bpr.2022.100083)
Supplement: Document S2. Article plus supporting material [file mmc2.pdf]

Biophysical Reports

REPORT

Permissive and nonpermissive channel closings in CFTR revealed by a factor graph inference algorithm

Alexander S. Moffett,[1] Guiying Cui,[2] Peter J. Thomas,[3] William D. Hunt,[4] Nael A. McCarty,[2] Ryan S. Westafer,[5] and Andrew W. Eckford[1,*]

[1]Department of Electrical Engineering and Computer Science, York University, Toronto, ON, Canada; [2]Emory + Children's Center for Cystic Fibrosis and Airways Disease Research, Emory University School of Medicine and Children's Healthcare of Atlanta, Atlanta, Georgia; [3]Department of Mathematics, Applied Mathematics, and Statistics, Case Western Reserve University, Cleveland, Ohio; [4]School of Electrical and Computer Engineering, Georgia Institute of Technology, Atlanta, Georgia; and [5]Georgia Tech Research Institute, Atlanta, Georgia

ABSTRACT The closing of the gated ion channel in the cystic fibrosis transmembrane conductance regulator can be categorized as nonpermissive to reopening, which involves the unbinding of ADP or ATP, or permissive, which does not. Identifying the type of closing is of interest as interactions with nucleotides can be affected in mutants or by introducing agonists. However, all closings are electrically silent and difficult to differentiate. For single-channel patch-clamp traces, we show that the type of the closing can be accurately determined by an inference algorithm implemented on a factor graph, which we demonstrate using both simulated and lab-obtained patch-clamp traces.

SIGNIFICANCE Membrane ion channels are embedded in the plasma membranes of many eukaryotic cells, and the current through these channels can be measured using a patch-clamp apparatus. The opening and closing of an ion channel are dependent on a sequence of conformational changes between structural states, called kinetic microstates. These microstates are crucial to understanding the dynamics of the channel and are a subject of intense theoretical and experimental interest. In cystic fibrosis transmembrane conductance regulator, ATP binding occurs only in certain sequences of state transitions, while toxins, inhibitors, and point mutations are known to have a direct impact on these transitions. However, experiments to directly observe hidden (i.e., electrically equivalent) states are complex, and challenges remain in characterizing the states in their dynamic context.

In contrast to experimental methods, Bayesian inference and the expectation-maximization algorithm are simple and well-known techniques which have long been used as a solution to hidden-variable inference problems, including patch-clamp traces. These techniques are combined on graphical models called factor graphs. Applying a factor graph inference algorithm to cystic fibrosis transmembrane conductance regulator ion currents, our algorithm accurately distinguishes between permissive channel closings (without unbinding of nucleotide) and nonpermissive closings (with unbinding of nucleotide), providing insight into an otherwise hidden, but physiologically important, process. Our method is flexible and can be used to complement or improve other contemporary analytical or experimental techniques.

INTRODUCTION

Cystic fibrosis (CF) is a life-threatening genetic disease affecting the respiratory and digestive systems that is caused by mutations to the CF transmembrane conductance regulator (CFTR) anion channel (1,2). CFTR is a “broken” member of the ATP-binding cassette transporter class in that CFTR acts as an ATP-gated ion channel rather than an active transporter as is the function of other ATP-binding cassette transporters. CFTR consists of a single polypeptide chain, with two transmembrane domain-nucleotide binding domain (NBD) pairs connected through a region called the R domain (3). Each of the two NBDs contribute to both of the two known binding sites for ATP, although only one of these sites facilitates the hydrolysis of ATP to ADP. The two transmembrane domains form a gated channel that is controlled by the state of the two intracellular NBDs.

Submitted May 14, 2022, and accepted for publication October 13, 2022.

*Correspondence: aeckford@yorku.ca

Alexander S. Moffett's present address is Center for Theoretical Biological Physics and Department of Physics, Northeastern University, Boston, Massachusetts

Editor: Sarah Rauscher.

https://doi.org/10.1016/j.bpr.2022.100083

© 2022 The Author(s).

This is an open access article under the CC BY license (http://creativecommons.org/licenses/by/4.0/).

Of key interest are permissive and nonpermissive closings of the CFTR ion channel, in the sense of permissive to rapid reopening the channel. Considering the kinetic model in Fig. 1 (see (4)), nonpermissive closings involve the release of ADP in order to enable the binding of ATP (5,6) and include the irreversible $C4 \rightarrow C1a$ transition. On the other hand, permissive closings do not include this transition, meaning that they do not involve the release of ADP or ATP, leading to faster reopening. However, as these transitions occur on states in the same conductance level, the two types of closing cannot be directly distinguished by the patch clamp. The problem addressed by this report is to distinguish permissive and nonpermissive closings solely by performing signal processing on the patch-clamp trace.

Hidden-variable problems of this type have a long history in biophysics, e.g., (7). More recently, methods have been developed to automate idealization of noisy ion channel current recordings, including the expectation-maximization (EM) algorithm (8) and deep-learning (9) approaches. Nonparametric Bayesian approaches have been used to identify the number of kinetically distinct hidden states (10). Finally, many methods have been developed for estimation of the hidden state transition matrix, including maximum likelihood methods (11–15) and Bayesian approaches (16–19). Bayesian inference has also been used to estimate hidden kinetic states from synthetic patch-clamp measurements in (16), using a Markov chain Monte Carlo method.

In this report, we are interested in factor-graph-based inference algorithms, such as the sum-product algorithm (20,21), which can combine inference with EM-based estimation of unknown parameters (22,23). Algorithms of this type have been used in biomedical hidden-variable problems (24). Distinct from (16), the factor graph approach calculates posterior probabilities efficiently (and exactly if parameters are known). Elsewhere, applications of inference algorithms are found in diverse areas such as bioinformatics (25,26), biophysics (27,28), telecommunications, and, more recently, in machine learning (29).

The main contribution of this report is to show that permissive and nonpermissive closings of CFTR (i.e., the timing of ADP dissociation and ATP binding) can be accurately inferred from patch-clamp traces alone, without any additional equipment, model training, or prior knowledge about parameter values. In doing so, we apply a factor-graph-based inference algorithm; to our knowledge, our report is also the first to apply an inference algorithm of this kind to CFTR.

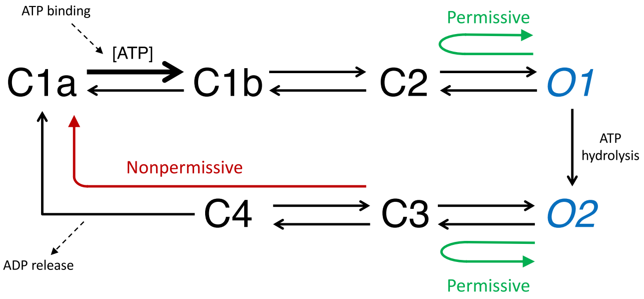

FIGURE 1 CFTR cycle model. Possible state transitions are indicated with black arrows. The C1a → C1b transition is sensitive to ATP concentration and is indicated with a bold arrow and is labeled [ATP]. States with open and closed ion channels are indicated in blue italics and black, respectively. Closings for which the reopening occurs in the same state are called permissive, depicted with green arrows; closings from O2 where the reopening is O1 are called nonpermissive, depicted with a red arrow. See also reference (4) and the supporting material.

MATERIALS AND METHODS

Receptor model

We use a physical model of CFTR described in Fig. 1 (see (4)). In this seven-state model, states C1a, C1b, C2, C3, and C4 are fully closed, so we assume that ions are completely unable to pass through when CFTR is in these states, and thus, they have the same conductance level. States O1 and O2 are open states in which an ion current can flow through the channel. (The states are labeled so that the first letter indicates whether the channel is closed [C] or open [O].) Beginning with C1a, with a single ATP bound at the first ATP binding site, which is incapable of hydrolysis (30), the reversible transition to C1b occurs when a second ATP binds so that both binding sites are occupied. Because this step involves ATP binding, the rate depends on the concentration of ATP. The reversible transitions from C1b to C2 and from C2 to O1 are conformational changes resulting in an open CFTR, much like any ligand-gated channel such as the acetylcholine receptor (31). The transition from O1 to O2 is the first of two irreversible steps in the cycle, with one NBD-bound ATP undergoing hydrolysis to ADP. CFTR can then undergo reversible conformational changes from O2 to C3 and from C3 to C4, resulting in a closed pore. Finally, the second irreversible step occurs in the transition from C4 to C1a, where the NBD-bound ADP unbinds from CFTR, leaving one apo and one filled ATP binding site. This seven-state model is in agreement with the four-state simplified cyclic gating model of (32) and models distinguishing multiple closed and/or open states (33,34).

As noted in the introduction, we are interested in determining whether the channel closings are permissive or nonpermissive. Nonpermissive closings include the irreversible C4 to C1a transition, in which ADP unbinds and a binding site is available for ATP, while permissive closings do not. Thus, considering Fig. 1,

- A permissive closing has the same initial and final open states, i.e. $O1 \rightarrow C2 \ldots C2 \rightarrow O1$ or $O2 \rightarrow C3 \ldots C3 \rightarrow O2$, and
- A nonpermissive closing has different initial and final open states, i.e., $O2 \rightarrow C3 \ldots C2 \rightarrow O1$. From Fig. 1, the only way to do this is to proceed through the $C4 \rightarrow C1a$ transition.

The states under the ellipsis $(\ldots)$ can be any valid sequence of closed states from Fig. 1, not necessarily the same state.

The kinetic microstates of CFTR can be modeled using a master equation of the form

$$\frac{dP}{dt} = PR, \quad (1)$$

where P is a row vector with length equal to the number of kinetic microstates and R is a square matrix of kinetic rates for each possible state transition. In this formulation, P_i is the probability that a receptor is in microstate i, while R_{ij} is the transition rate from state i to state j. The rate matrix R and the full master equation are given in the supporting material.

Patch-clamp signal model

Formally, our system contains a set $\mathcal{V}$ of observable conductance states, a set $\mathcal{S}$ of hidden kinetic microstates, and a mapping $m : \mathcal{S} \to \mathcal{V}$ from microstates to conductance states. For CFTR, we have

$$\mathcal{V} = \{0, 1\}, \tag{2}$$

$$\mathcal{S} = \{\mathrm{C1a}, \mathrm{C1b}, \mathrm{C2}, \mathrm{C3}, \mathrm{C4}, \mathrm{O1}, \mathrm{O2}\}, \text{ and} \tag{3}$$

$$m(s) = \begin{cases} 0, & s \in \{\mathrm{C1a}, \mathrm{C1b}, \mathrm{C2}, \mathrm{C3}, \mathrm{C4}\} \\ 1, & s \in \{\mathrm{O1}, \mathrm{O2}\} \end{cases}. \tag{4}$$

The conductance states $\{0, 1\}$ correspond to the ion channel's current when closed and open, respectively.

The patch clamp observes the channel current through additive noise and samples these observations with sampling time Δt to form discrete-time signals. Let $y = [y_1, y_2, \ldots, y_n] \in \mathbb{R}^n$ represent the sequence of observations for a single channel, and let $s = [s_1, s_2, \ldots, s_n] \in \mathcal{S}^n$ represent the corresponding microstates. Then, at the k th sample, the patch clamp measures

$$y_k = I_{s_k} + n_k, \tag{5}$$

where $I_{s_k} = Am(s_k)$ is the current flowing through the channel in state s_k, given by a constant A and the function $m(s)$ in (4), and where n_k forms a sequence of independent, identically distributed Gaussian random variables with zero mean and variance σ^2. Using the central limit theorem, it is reasonable to model the noise as Gaussian, particularly if a decimation filter is applied to the raw patch-clamp outputs (see the results section below).

The transitions of microstates $s_{k-1} \to s_k$ are modeled as a discrete-time Markov chain (35), again with a discrete time step Δt. The transition probability matrix for this Markov chain $Q = [Q_{ij}] = [\Pr(s_k = j \mid s_{k-1} = i)]$ is given by the solution to (1):

$$Q = e^{R\Delta t}. \tag{6}$$

Inference, parameter estimation, and simulation

We use the sum-product algorithm (21) over the factor graph representing the sequence of states s to obtain the a posteriori distribution $p(s_k \mid y)$. Meanwhile, the transition probability matrix Q and noise variance σ^2 (and potentially the current amplitude A) are unknown a priori and must be estimated from the data. The EM algorithm (36) is a standard tool for this kind of simultaneous inference-estimation task. We employ a variant of the EM algorithm, known as the factor graph EM algorithm (22,37), which is intended for use alongside sum-product inference algorithms. The complete details of our algorithm are described in the supporting material.

Given $p(s_k \mid y)$, we define a confidence threshold C, $0 \leq C < 1$ and use the following decision rule to obtain the estimate $\hat{s}_k$ for each s_k:

$$\hat{s}_k = \begin{cases} \mathrm{argmax}_{s_k \in \mathcal{S}} p(s_k \mid y), & p(s_k \mid y) > C, \\ \varnothing, & \text{otherwise.} \end{cases} \tag{7}$$

That is, $\hat{s}$ is the maximum a posteriori (MAP) estimate if the estimate exceeds C; otherwise, $\hat{s}$ is null $(\varnothing)$. Setting $C = 0$ obtains the MAP estimate for all states s_k, while setting $C > 0$ reduces the probability of false alarm.

We test our inference algorithm via Monte Carlo simulation by generating instances of discrete-time Markov chains with transition probability matrix Q (Eq. 6) and adding noise (Eq. 5). To evaluate the algorithm, we consider probability of false alarm P_{FA} and probability of missed detection P_{MD}, also known, respectively, as type I and type II errors. First, using the ground-truth state sequence s_k, we make a list L_{GT} of closings (i.e., any sequence OX→CX… CX→OX, with OX and CX representing any open or closed state, respectively, and where all states under the ellipsis $(\ldots)$ are closed states), and a list $L_{\mathrm{GT}}^{(\mathrm{np})}$ of nonpermissive closings. Next, using the sequence of estimated states $\hat{s}_k$, we make similar lists of all estimated closings L_{E} and estimated nonpermissive closings $L_{\mathrm{E}}^{(\mathrm{np})}$. Represent the length of a list as, e.g., $|L_{\mathrm{GT}}|$. Now, let n_{FA} be the number of closings that appear in $L_{\mathrm{E}}^{(\mathrm{np})}$ but not in $L_{\mathrm{GT}}^{(\mathrm{np})}$ and let n_{MD} be the number of closings that appear in $L_{\mathrm{GT}}^{(\mathrm{np})}$ but not in $L_{\mathrm{E}}^{(\mathrm{np})}$. Then,

$$P_{\mathrm{FA}} = \frac{n_{\mathrm{FA}}}{\left|L_{\mathrm{E}}^{(\mathrm{np})}\right|}, \; P_{\mathrm{MD}} = \frac{n_{\mathrm{MD}}}{\left|L_{\mathrm{GT}}^{(\mathrm{np})}\right|}. \tag{8}$$

Patch-clamp measurements

Single CFTR channels were studied in inside-out patches pulled from *Xenopus* oocytes injected with cRNA encoding the wild-type channel, as previously described in (38). Briefly, to enable removal of the vitelline membrane, oocytes were placed in a bath solution containing (in mM) 200 monopotassium aspartate, 20 KCl, 1 $MgCl_2$, 10 EGTA, and 10 HEPES (pH 7.2) adjusted with KOH. Gigaohm seals were formed with patch pipettes pulled from borosilicate glass and filled with solution containing (in mM) 150 N-methyl-D-glutamine chloride, 5 $MgCl_2$, and 10 TES buffer (pH 7.5). After excision of the patch,

TABLE 1 Parameters for the CFTR channel, corresponding to the model in Figure 1

	Destination state						
Origin state	C1a	C1b	C2	O1	O2	C3	C4
C1a		$9.0 \cdot 10^3$ (M s)$^{-1}$ [ATP]					
C1b	5.0 s^{-1}		7.7 s^{-1}				
C2		5.8 s^{-1}		4.9 s^{-1}			
O1			10.0 s^{-1}		7.1 s^{-1}		
O2						3.0 s^{-1}	
C3					7.0 s^{-1}		6.0 s^{-1}
C4	1.7 s^{-1}					12.8 s^{-1}	

Parameters are derived to match the wild-type (high $\boldsymbol{P_o}$) model from (4). A blank entry indicates that the transition is impossible. [ATP] indicates molar concentration of ATP.

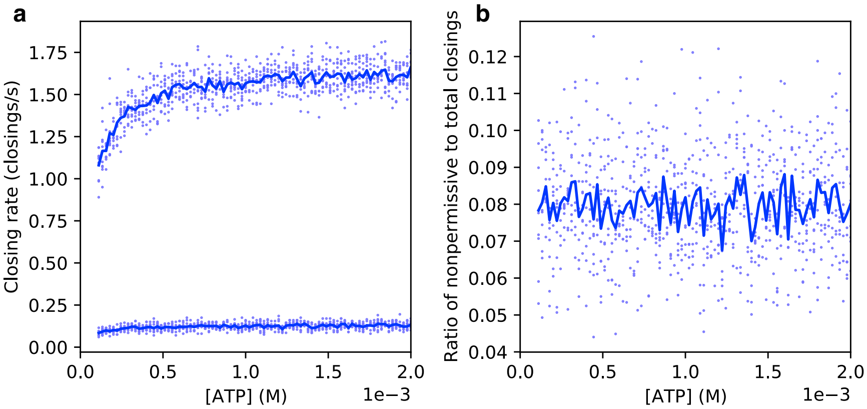

FIGURE 2 Prevalence of nonpermissive closings. (*a*) Rate of all channel closings (*top line*) and nonpermissive closings (*bottom line*) versus ATP concentration. (*b*) Ratio of nonpermissive to total closings versus ATP concentration. Dots represent the outcomes of each simulation run, and lines represent mean value at each concentration. Sampling rate = 100 Hz; rate parameters from Table 1.

CFTR channels were activated by bath solution containing 150 mM N-methyl-D-glutamine chloride, 1.1 mM $MgCl_2$, 2 mM Tris-EGTA, 10 mM TES buffer, 1 mM MgATP, and 127 U/ml PKA (pH 7.5). Currents were recorded at $V_M = -100$ mV using an Axopatch 200B amplifier, with filtering at 0.1–1 kHz. The sampling rate of the patch clamp data was 2 kHz.

Code

Code and raw patch-clamp traces used to generate all results in this report are publicly available on Zenodo (see (39)). We do not include simulated patch-clamp traces in this dataset, but simulations can be generated using the code we provide. The code repository also includes a Jupyter notebook and raw data for generating all results in this report.

RESULTS

Analysis using simulated patch-clamp measurements

Here, we give results obtained from Monte Carlo simulations of the CFTR ion channel. To generate simulated

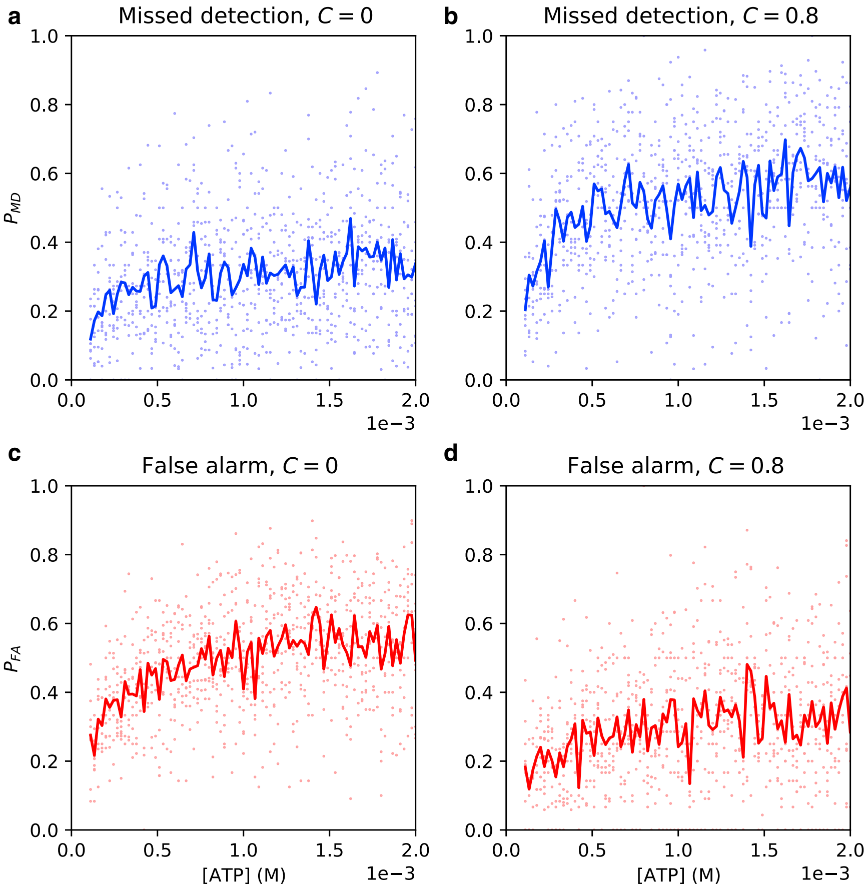

FIGURE 3 Missed detection and false alarm probabilities. (*a* and *b*) Missed detection probability versus ATP concentration for confidence thresholds $\boldsymbol{C} = \mathbf{0}$ and $\boldsymbol{C} = \mathbf{0.8}$, respectively. (*c* and *d*) False alarm probability versus ATP concentration for $C = 0$ and $\boldsymbol{C} = \mathbf{0.8}$, respectively. Dots represent each simulation run, while lines represent the average at each concentration. Sampling rate = 100 Hz, 20,000 samples, 400 EM iterations, $\boldsymbol{\sigma^2} = \mathbf{0.02}$; rate parameters from Table 1.

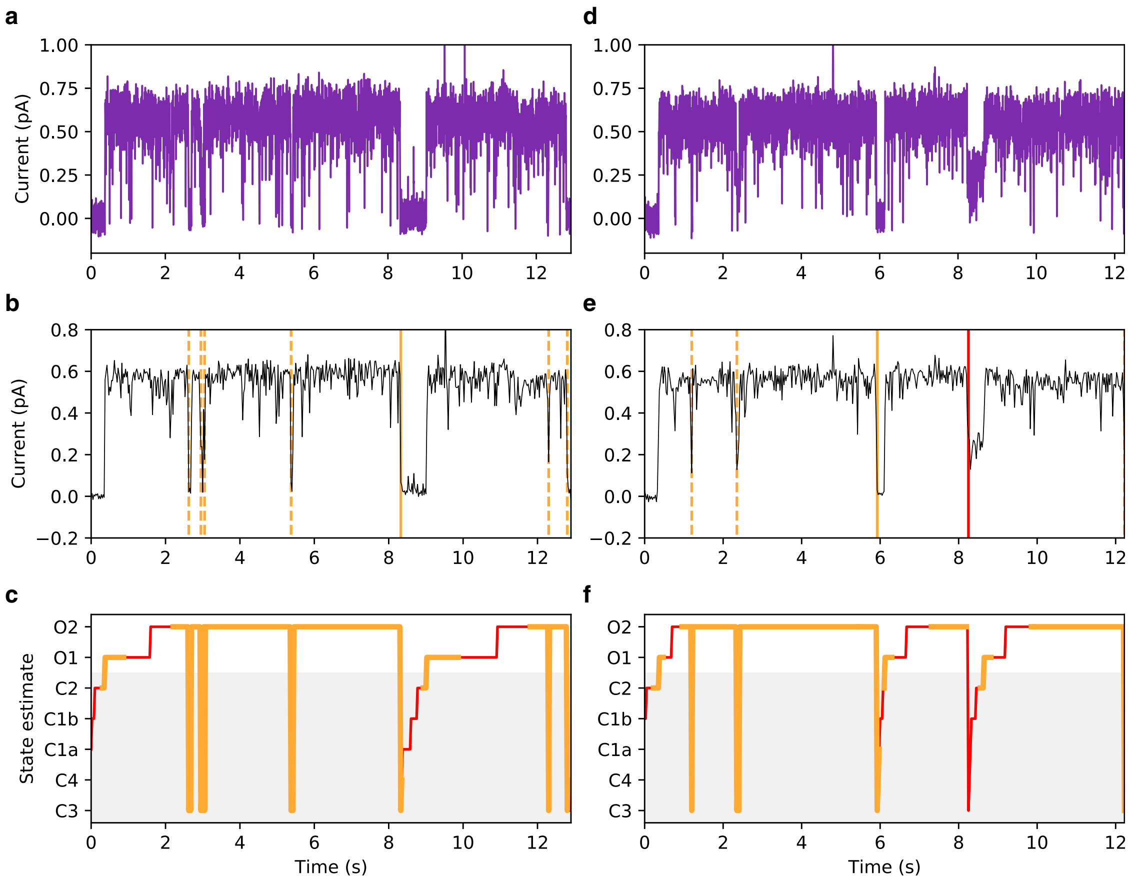

FIGURE 4 CFTR patch-clamp measurements (raw and preprocessed) along with the corresponding inferred states for two different experiments, one in each column. (*a* and *d*, *top row*) Measured patch-clamp current. (*b* and *e*, *middle row*) Patch-clamp current signal after decimation; this signal is provided to the inference algorithm. Detected closings are depicted on these figures, with dashed/solid vertical lines, respectively, indicating permissive/nonpermissive closings; orange/red lines represent detected closings that, respectively, exceed/do not exceed $\boldsymbol{C} = \mathbf{0.8}$. (*c* and *f*, *bottom row*) Inferred state after 400 EM iterations; orange/red lines represent state estimates that, respectively, exceed/do not exceed $\boldsymbol{C} = \mathbf{0.8}$.

patch-clamp results, we use example model parameters for the seven-state CFTR model given in Table 1. These example values are not provided to the inference algorithm, so performance does not in general depend on their accuracy.

Properties of nonpermissive closings

In Fig. 2, we used our simulator to generate ground-truth state sequences s_k; we then count the number of nonpermissive closings $\left|L_{\mathrm{GT}}^{(\mathrm{np})}\right|$ and the total number of closings $|L_{\mathrm{GT}}|$ and divide by time to get rates. In the figure, we see that nonpermissive closings occur at a much lower rate than permissive closings. While the rate of closings depends on ATP concentration due to the cyclical nature of CFTR gating, the ratio of nonpermissive to total closings remains nearly independent of concentration. These observations are consistent with the dynamics of our model: a low ATP concentration will lead to a longer dwell time in state C1a, thus increasing the interval between openings without affecting the rates of transitions at the boundary between open and closed. Moreover, nonpermissive closings are relatively rare, with a ratio of roughly 1 nonpermissive closing per 12 total closings.

Missed detection and false alarm probability

In Fig. 3, we give missed detection and false alarm probabilities, P_{MD} and P_{FA}, using our algorithm (see Eq. 8). Setting the confidence threshold $C = 0$ gives the MAP estimate of each state (see Eq. 7); we see a low P_{MD}, meaning few nonpermissive closings are missed, but P_{FA} is relatively high, i.e., around 50%. (Since nonpermissive closings are rare, cf. Fig. 2 *b*; this is still far better than random guessing.) With a higher confidence threshold of $C = 0.8$, P_{FA} is reduced at the expense of increased P_{MD}. This is explained by noting that lower-confidence state estimates are discarded, so those closings will be missed by the algorithm. This demonstrates that C can be adjusted to

trade off P_{FA} against P_{MD}. The performance of the algorithm is dependent on ATP concentration, with error rates increasing as concentration increases.

Application to CFTR mutants

We modified the rate matrix in Table 1 to represent the K1250A CFTR mutant (see (4)). Complete details and results, similar to Figs. 2 and 3, are provided in the supporting material.

Application to experimentally obtained patch-clamp measurements

In Fig. 4, we show the application of our algorithm to lab-obtained CFTR patch-clamp measurements. We show two examples corresponding to two different experiments. For techniques used to obtain these measurements, see the materials and methods section.

As the patch-clamp signal is oversampled compared with the kinetics of CFTR, we decimate the signal by a factor of 50, applying a block-averaging decimation filter (taking the sample average over nonoverlapping blocks of 50 samples). The decimation step is performed to reduce the noise at high frequencies, which contains very little useful information about the signal, while preserving the features of interest at lower frequencies, improving the performance of the algorithm. We show the decimated signal in the middle plots of Fig. 4, overlaid with vertical lines indicating the closings found by our algorithm, both permissive and nonpermissive. In the bottom plots of Fig. 4, we give the state estimates $\hat{s}_k$ found by the algorithm, with different colors indicating whether or not the initial and final estimated states both exceed a confidence threshold of $C = 0.8$. From the raw data and preprocessed traces, abrupt transitions from high to low current correspond well with the detected closings.

DISCUSSION

We show that an algorithmic tool reveals the precise microstate kinetics of CFTR. By revealing permissive and nonpermissive closings, we can precisely estimate the timing of each nucleotide unbinding event, a key step in CFTR's kinetic model. Furthermore, our method may be used to study the effect of reagents that are known to affect hidden-state kinetics of CFTR, such as scorpion venom (4); future experiments in this direction might also be used to analyze pharmaceuticals that target CFTR. More generally, beyond permissive and nonpermissive closings, this method gives the designer of an experiment a novel and fine-grained algorithmic tool to discover changes to the behavior of receptor proteins. For example, this method could be used to determine the fine-grained, microstate-by-microstate effects of particular agonists or mutations, in CFTR or other receptors.

SUPPORTING MATERIAL

Supporting material can be found online at https://doi.org/10.1016/j.bpr.2022.100083.

AUTHOR CONTRIBUTIONS

A.S.M., N.A.M., and A.W.E. wrote the article. A.W.E. designed the research, wrote the simulation code, and carried out the simulations. N.A.M. and G.C. performed the patch clamp experiments. P.J.T., W.D.H., and R.S.W. edited the article and contributed research ideas.

ACKNOWLEDGMENTS

A.S.M. and A.W.E. were funded by DARPA via the RadioBio program under grant number HR001117C0125. P.J.T. was supported by NSF via grant number DMS-2052109. W.D.H., N.A.M., and R.S.W. were funded in part by DARPA via the RadioBio program under grant number HR001117C0124.

P.J.T. acknowledges Oberlin College Libraries for research assistance.

DECLARATION OF INTERESTS

The authors declare no competing interests.

REFERENCES

1. Csanády, L., P. Vergani, and D. C. Gadsby. 2019. Structure, gating, and regulation of the CFTR anion channel. *Physiol. Rev.* 99:707–738.
2. Rey, M. M., M. P. Bonk, and D. Hadjiliadis. 2019. Cystic fibrosis: Emerging understanding and therapies. *Annu. Rev. Med.* 70:197–210.
3. Zhang, Z., and J. Chen. 2016. Atomic structure of the cystic fibrosis transmembrane conductance regulator. *Cell.* 167:1586–1597.e9.
4. Fuller, M. D., Z.-R. Zhang, …, N. A. McCarty. 2005. The block of CFTR by scorpion venom is state-dependent. *Biophys. J.* 89:3960–3975.
5. Gout, T. 2012. Role of ATP binding and hydrolysis in the gating of the cystic fibrosis transmembrane conductance regulator. *Ann. Thorac. Med.* 7:115–121.
6. Zhang, Z., F. Liu, and J. Chen. 2018. Molecular structure of the ATP-bound, phosphorylated human CFTR. *Proc. Natl. Acad. Sci. USA.* 115:12757–12762.
7. Colquhoun, D., and A. G. Hawkes. 1981. On the stochastic properties of single ion channels. *Proc. R. Soc. Lond. B Biol. Sci.* 211:205–235.
8. Shah, S. I., A. Demuro, …, G. Ullah. 2018. TraceSpecks: a software for automated idealization of noisy patch-clamp and imaging data. *Biophys. J.* 115:9–21.
9. Celik, N., F. O'Brien, …, R. Barrett-Jolley. 2020. Deep-Channel uses deep neural networks to detect single-molecule events from patch-clamp data. *Commun. Biol.* 3:3–10.

10. Hines, K. E., J. R. Bankston, and R. W. Aldrich. 2015. Analyzing single-molecule time series via nonparametric Bayesian inference. *Biophys. J.* 108:540–556.

11. Qin, F., A. Auerbach, and F. Sachs. 1996. Estimating single-channel kinetic parameters from idealized patch-clamp data containing missed events. *Biophys. J.* 70:264–280.

12. Colquhoun, D., A. Hawkes, and K. Srodzinski. 1996. Joint distributions of apparent open and shut times of single-ion channels and maximum likelihood fitting of mechanisms. *Philos. Trans. Royal Soc. A.* 354:2555–2590.

13. Colquhoun, D., C. J. Hatton, and A. G. Hawkes. 2003. The quality of maximum likelihood estimates of ion channel rate constants. *J. Physiol.* 547:699–728.

14. Moffatt, L. 2007. Estimation of ion channel kinetics from fluctuations of macroscopic currents. *Biophys. J.* 93:74–91.

15. Nicolai, C., and F. Sachs. 2013. Solving ion channel kinetics with the QuB software. *Biophys. Rev. Lett.* 08:191–211.

16. Rosales, R., J. A. Stark, …, S. B. Hladky. 2001. Bayesian restoration of ion channel records using hidden Markov models. *Biophys. J.* 80:1088–1103.

17. Gin, E., M. Falcke, …, J. Sneyd. 2009. Markov chain Monte Carlo fitting of single-channel data from inositol trisphosphate receptors. *J. Theor. Biol.* 257:460–474.

18. Siekmann, I., L. E. Wagner, II, …, J. Sneyd. 2011. MCMC estimation of Markov models for ion channels. *Biophys. J.* 100:1919–1929.

19. Epstein, M., B. Calderhead, …, L. G. Sivilotti. 2016. Bayesian statistical inference in ion-channel models with exact missed event correction. *Biophys. J.* 111:333–348.

20. MacKay, D. 2003. Information Theory, Inference and Learning Algorithms. Cambridge University Press, Cambridge.

21. Kschischang, F. R., B. J. Frey, and H.-A. Loeliger. 2001. Factor graphs and the sum-product algorithm. *IEEE Trans. Inf. Theor.* 47:498–519.

22. Dauwels, J., A. Eckford, …, H.-A. Loeliger. 2009. Expectation maximization as message passing-Part I: Principles and Gaussian Messages. Preprint at arXiv. https://doi.org/10.1101/arXiv.0910.2832.

23. Eckford, A. W. 2005. The factor graph EM algorithm: Applications for LDPC codes. *In* IEEE 6th Workshop on Signal Processing Advances in Wireless Communications. IEEE, pp. 910–914.

24. Wadehn, F. 2019. State space methods with applications in biomedical signal processing. ETH Zurich, Zurich.

25. Xiong, H. Y., Y. Barash, and B. J. Frey. 2011. Bayesian prediction of tissue-regulated splicing using RNA sequence and cellular context. *Bioinformatics.* 27:2554–2562.

26. Meyer, X., L. Dib, …, N. Salamin. 2019. Simultaneous Bayesian inference of phylogeny and molecular coevolution. *Proc. Natl. Acad. Sci. USA.* 116:5027–5036.

27. Metzner, P., F. Noé, and C. Schütte. 2009. Estimating the sampling error: Distribution of transition matrices and functions of transition matrices for given trajectory data. *Phys. Rev. E - Stat. Nonlinear Soft Matter Phys.* 80:021106.

28. Potrzebowski, W., J. Trewhella, and I. Andre. 2018. Bayesian inference of protein conformational ensembles from limited structural data. *PLoS Comput. Biol.* 14:e1006641.

29. Barber, D. 2012. Bayesian reasoning and machine learning. Cambridge University Press, Cambridge.

30. Infield, D. T., K. M. Strickland, …, N. A. McCarty. 2021. The molecular evolution of function in the CFTR chloride channel. *J. Gen. Physiol.* 153:e202012625.

31. Albuquerque, E. X., E. F. R. Pereira, …, S. W. Rogers. 2009. Mammalian nicotinic acetylcholine receptors: From structure to function. *Physiol. Rev.* 89:73–120.

32. Csanády, L., and B. Töröcsik. 2014. Catalyst-like modulation of transition states for CFTR channel opening and closing: New stimulation strategy exploits nonequilibrium gating. *J. Gen. Physiol.* 143:269–287.

33. Vergani, P., S. W. Lockless, …, D. C. Gadsby. 2005. CFTR channel opening by ATP-driven tight dimerization of its nucleotide-binding domains. *Nature.* 433:876–880.

34. Csanády, L. 2017. CFTR gating: Invisible transitions made visible. *J. Gen. Physiol.* 149:413–416.

35. Smith, G. D. 2002. Modeling the stochastic gating of ion channels. *In* Computational cell biology. Springer, pp. 285–319.

36. Dempster, A. P., N. M. Laird, and D. B. Rubin. 1977. Maximum likelihood from incomplete data via the EM algorithm. *J. Roy. Stat. Soc. B.* 39:1–22.

37. Loeliger, H.-A., J. Dauwels, …, F. R. Kschischang. 2007. The factor graph approach to model-based signal processing. *Proc. IEEE.* 95:1295–1322.

38. Infield, D. T., G. Cui, …, N. A. McCarty. 2016. Ion Channels and Transporters in Lung Function and Disease: Positioning of extracellular loop 1 affects pore gating of the cystic fibrosis transmembrane conductance regulator. *Am. J. Physiol. Lung Cell Mol. Physiol.* 310:L403–L414.

39. Eckford, A. 2022. andreweckford/PatchClampFactorGraphEM: Archival release 1. https://doi.org/10.5281/zenodo.7073043.

Biophysical Reports, Volume *2*

Supplemental information

Permissive and nonpermissive channel closings in CFTR revealed by a factor graph inference algorithm

Alexander S. Moffett, Guiying Cui, Peter J. Thomas, William D. Hunt, Nael A. McCarty, Ryan S. Westafer, and Andrew W. Eckford

Supplemental Information

Equation references of the form (1) refer to equations in the main paper, while references of the form (S1) refer to equations in this supplemental information.

1 Rate matrix

The transition rates are given in the rate matrix (with rows and columns, in order, corresponding to C1a, C1b, C2, O1, O2, C3, and C4):

$$R = \begin{bmatrix} R_{11} & k_{\mathrm{C1a}\to\mathrm{C1b}} & 0 & 0 & 0 & 0 & 0 \\ k_{\mathrm{C1b}\to\mathrm{C1a}} & R_{22} & k_{\mathrm{C1b}\to\mathrm{C2}} & 0 & 0 & 0 & 0 \\ 0 & k_{\mathrm{C2}\to\mathrm{C1b}} & R_{33} & k_{\mathrm{C2}\to\mathrm{O1}} & 0 & 0 & 0 \\ 0 & 0 & k_{\mathrm{O1}\to\mathrm{C2}} & R_{44} & k_{\mathrm{O1}\to\mathrm{O2}} & 0 & 0 \\ 0 & 0 & 0 & 0 & R_{55} & k_{\mathrm{O2}\to\mathrm{C3}} & 0 \\ 0 & 0 & 0 & 0 & k_{\mathrm{C3}\to\mathrm{O2}} & R_{66} & k_{\mathrm{C3}\to\mathrm{C4}} \\ k_{\mathrm{C4}\to\mathrm{C1a}} & 0 & 0 & 0 & 0 & k_{\mathrm{C4}\to\mathrm{C3}} & R_{77} \end{bmatrix} \tag{S1}$$

with the diagonal entries R_{ii} set so that each row of R sums to zero, and with $R_{ij} = 0$ indicating that the transition $i \to j$ is forbidden. Table 1 gives example values for each rate, noting that the value of $k_{\mathrm{C1a}\to\mathrm{C1b}}$ is dependent on the environmental concentration of ATP.

2 Master equation

Consider the master equation from equation (1) in the main paper. Here P gives the occupancy probabilities for each microstate. In the case of the CFTR receptor,

$$P = \Big[P(\mathrm{C1a}), P(\mathrm{C1b}), P(\mathrm{C2}), P(\mathrm{O1}), P(\mathrm{O2}), P(\mathrm{C3}), P(\mathrm{C4})\Big]. \tag{S2}$$

Using (S2) and (S1), the continuous-time master equation is fully expanded by

$$\frac{dP(\mathrm{C1a})}{dt} = -k_{\mathrm{C1a}\to\mathrm{C1b}}P(\mathrm{C1a}) + k_{\mathrm{C4}\to\mathrm{C1a}}P(\mathrm{C4}) + k_{\mathrm{C1b}\to\mathrm{C1a}}P(\mathrm{C1b}) \tag{S3}$$

$$\frac{dP(\mathrm{C1b})}{dt} = -(k_{\mathrm{C1b}\to\mathrm{C2}} + k_{\mathrm{C1b}\to\mathrm{C1a}})P(\mathrm{C1b}) + k_{\mathrm{C1a}\to\mathrm{C1b}}P(\mathrm{C1a}) + k_{\mathrm{C2}\to\mathrm{C1b}}P(\mathrm{C2}) \tag{S4}$$

$$\frac{dP(\mathrm{C2})}{dt} = -(k_{\mathrm{C2}\to\mathrm{O1}} + k_{\mathrm{C2}\to\mathrm{C1b}})P(\mathrm{C2}) + k_{\mathrm{C1b}\to\mathrm{C2}}P(\mathrm{C1a}) + k_{\mathrm{O1}\to\mathrm{C2}}P(\mathrm{O1}) \tag{S5}$$

$$\frac{dP(\mathrm{O1})}{dt} = -(k_{\mathrm{O1}\to\mathrm{O2}} + k_{\mathrm{O1}\to\mathrm{C2}})P(\mathrm{O1}) + k_{\mathrm{C2}\to\mathrm{O1}}P(\mathrm{C2}) \tag{S6}$$

$$\frac{dP(\mathrm{O2})}{dt} = -k_{\mathrm{O2}\to\mathrm{C3}}P(\mathrm{O2}) + k_{\mathrm{O1}\to\mathrm{O2}}P(\mathrm{O1}) + k_{\mathrm{C3}\to\mathrm{O2}}P(\mathrm{C3}) \tag{S7}$$

$$\frac{dP(\mathrm{C3})}{dt} = -(k_{\mathrm{C3}\to\mathrm{C4}} + k_{\mathrm{C3}\to\mathrm{O2}})P(\mathrm{C3}) + k_{\mathrm{O2}\to\mathrm{C3}}P(\mathrm{O2}) + k_{\mathrm{C4}\to\mathrm{C3}}P(\mathrm{C4}) \tag{S8}$$

$$\frac{dP(\mathrm{C4})}{dt} = -(k_{\mathrm{C4}\to\mathrm{C1a}} + k_{\mathrm{C4}\to\mathrm{C3}})P(\mathrm{C4}) + k_{\mathrm{C3}\to\mathrm{C4}}P(\mathrm{C3}). \tag{S9}$$

Example values of each rate are given in Table 1 in the main paper. We suppress the dependence of $k_{\mathrm{C1a}\to\mathrm{C1b}}$ on ATP concentration in our notation for the sake of compactness, but as noted in Table 1 the effects of ATP concentration are fully considered in the model.

3 Factor graphs and the sum-product algorithm

Factor graph inference is a method of probabilistic analysis. Let s be a random variable, which is of interest but cannot be directly observed, such as the kinetic microstates of CFTR. Let y be a collection of random variables that are observed, and correlated with s through a conditional probability $p(y \mid s)$; for example, y could be a collection of partial and/or noisy observations of s, such as the CFTR patch clamp measurements in this paper. Then the goal of factor graph inference is to calculate the conditional probability $p(s \mid y)$ of s given all knowledge y using Bayes' rule, i.e.,

$$p(s \mid y) = \frac{p(y \mid s)p(s)}{\sum_s p(y \mid s)p(s)}. \tag{S10}$$

The quantity $p(s \mid y)$ is known as the *a posteriori* probability, as it is calculated "after" observing y, and includes all available evidence about s. (This is as opposed to $p(s)$, called the *a priori* probability, which is our belief about s "before" observing any evidence y.) If we want to guess the value of s, then picking the value that maximizes $p(s|y)$, i.e. $\arg\max_s p(s|y)$, is known to be the decision rule with the smallest probability of error, and is called the maximum *a posteriori* (MAP) estimate.

The simplicity of (S10) belies the high computational complexity of this operation, particularly for large probabilistic models. Thus, efficient algorithms such as the sum-product algorithm are highly desirable. For more details and generalizations, the reader is encouraged to consult [1].

In CFTR, we observe noisy patch clamp currents $y = [y_1, y_2, \ldots, y_n]$, and we want to infer the underlying kinetic states $s = [s_1, s_2, \ldots, s_n]$. The sum-product algorithm for this problem is derived as follows. The probability mass function of the state sequence s can be written

$$p(s) = \prod_{k=1}^{n} p(s_k \mid s_{k-1}), \tag{S11}$$

where s_0 is null, i.e., $p(s_1 \mid s_0) = p(s_1)$. Including the noisy current observations y in the model, we can first write

$$p(y_k \mid s_k) = \frac{1}{\sqrt{2\pi\sigma^2}} \exp\left(-\frac{(y_k - I_{s_k})^2}{2\sigma^2}\right). \tag{S12}$$

where I_{s_k} is the current through the channel in state s_k. Finally, we have the joint probability mass function

$$p(y, s) = \prod_{k=1}^{n} p(y_k \mid s_k) p(s_k \mid s_{k-1}). \tag{S13}$$

The stochastic model $p(y, s)$ can be represented on a *factor graph*, where nodes representing variables $s_1, \ldots, s_n$ and $y_1, \ldots, y_n$ are connected to nodes representing factors in (S13), and an edge is drawn from variable to factor if the factor is a function of the variable. The factor graph for (S13) is depicted in Figure S1.

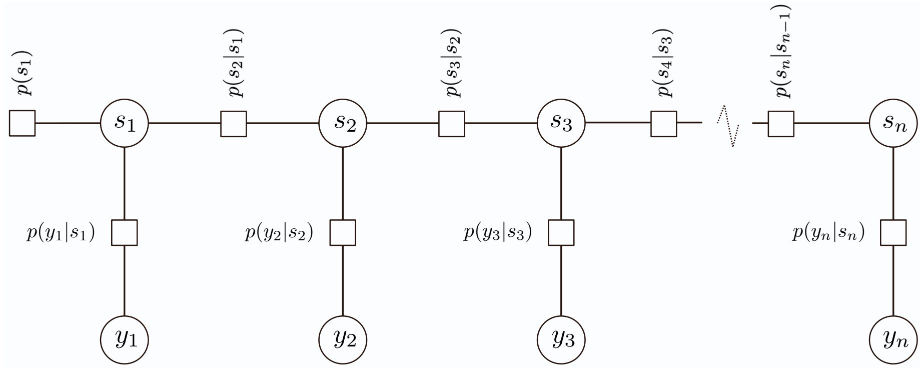

Figure S1: Factor graph for the patch clamp observations.

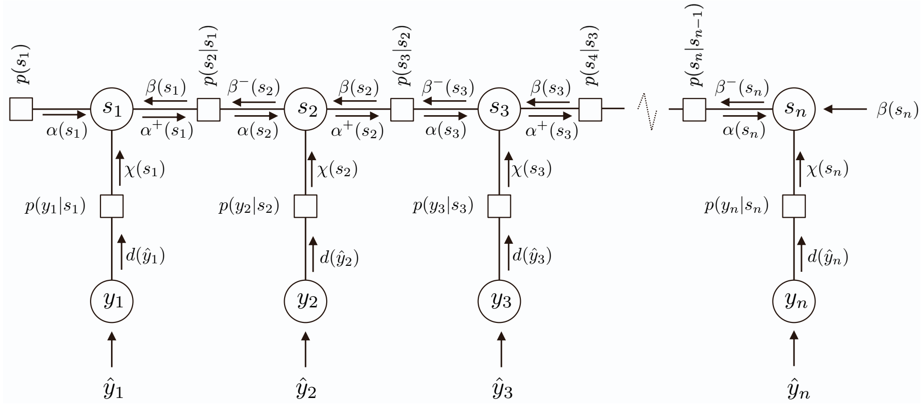

Figure S2: Factor graph for the patch clamp observations with sum-product messages indicated on each edge (cf. Figure S1).

We are interested in the *a posteriori* probability $p(s_k \mid y)$, i.e., the distribution over s_k knowing the observed (noisy) current for all time. This can be calculated using the forward-backward algorithm, a special case of the sum-product algorithm, which is a message-passing algorithm over the factor graph.

The flow of messages within the factor graph is depicted in Figure S2. *Generally speaking, messages on the outgoing edges from a node are calculated using all incoming messages to that node,* ***except*** *the incoming message along the same outgoing edge.*

In terms of the variable nodes s_k, the forward message $\alpha_v^+(s_k)$ is the component-wise product of the incoming forward message $\alpha_v(s_k)$ and the channel message $\chi(s_k)$ (defined below); the backward message $\beta_v^-(s_k)$ is the component-wise product of the incoming backward message $\beta_v(s_k)$ and the channel message $\chi(s_k)$ (defined below). It will be convenient to express the forward messages α as row vectors, and the backward messages β as column vectors. Representing these message calculations as matrix multiplications,

$$\alpha_v^+(s_k) = \alpha_v(s_k)\mathrm{diag}\Big(\chi(s_k)\Big) \tag{S14}$$

$$\beta_v^-(s_k) = \mathrm{diag}\Big(\chi(s_k)\Big)\beta_v(s_k) \tag{S15}$$

In terms of the factor nodes $p(y_k \mid s_k)$, $p(s_k \mid s_{k-1})$, each factor node "contains" the factor, and this factor is incorporated into the message calculation. As the factors are conditional probability functions in two variables, it will be convenient to represent the factors as matrices, with the conditional variable remaining constant along the rows, and the probabilistic variable remaining constant along the columns; this implies the matrices are row-stochastic. Message calculations are performed as follows:

- At $p(y_k \mid s_k)$, the channel message $\chi(s_k)$ is the vector

$$\chi(s_k) = \Big[\chi(s_k = 1), \chi(s_k = 2), \ldots, \chi(s_k = |\mathcal{S}|)\Big] \tag{S16}$$

  with slight abuse of the notation: $s_k = i$ means s_k is the ith element of $\mathcal{S}$

from (3). Moreover,

$$\chi(s_k = i) = p(y_k \mid s_k = i), \tag{S17}$$

where $p(y_k \mid s_k = i)$ is given by (S12). (For clarity, to obtain this value we substitute the patch clamp current observation y_k into (S17); with y_k and $s_k = i$ both defined, the result is a scalar constant.)

- At $p(s_k \mid s_{k-1})$, we have an outgoing forward message $\alpha(s_k)$ and an outgoing backward message $\beta(s_{k-1})$, with an incoming forward message $\alpha^+(s_{k-1})$ and incoming backward message $\beta^-(s_k)$. The internal factor $p(s_k \mid s_{k-1})$ is given by the transition probability matrix Q. The message calculation is given by

$$\alpha(s_k) = \alpha^+(s_{k-1})Q \tag{S18}$$

$$\beta(s_{k-1}) = Q\beta^-(s_k) \tag{S19}$$

Combining (S14) and (S18), and (S15) and (S19), we can write

$$\alpha(s_k) = \alpha(s_{k-1})\mathrm{diag}\Big(\chi(s_{k-1})\Big)Q \tag{S20}$$

$$\beta(s_k) = Q\mathrm{diag}\Big(\chi(s_{k+1})\Big)\beta(s_{k+1}) \tag{S21}$$

Next we must specify the order in which operations occur, i.e., the message-passing schedule:

- The channel messages $\chi(s_k)$ are precalculated.
- For the forward messages, the initial forward message $\alpha(s_1)$ is the steady-state distribution associated with Q. We then iteratively calculate (S20) for each $k = 2, 3, \ldots$.
- For the backward messages, we set the initial backward message $\beta(s_n) = [1, 1, \ldots, 1]$ (this message is uninformative, as the future gives us no information). We then iteratively calculate (S21) for each $k = n-1, n-2, \ldots$.

Finally, to calculate the *a posteriori* probability $p(s_k \mid y)$, we perform the complete message-passing schedule described above. Following the message calculations, we take the product of all messages inbound to variable node s_k, and normalize (to force the product to sum to 1). Letting $\odot$ represent component-wise multiplication:

$$p(s_k \mid y) = \frac{\alpha(s_k) \odot \beta(s_k) \odot \chi(s_k)}{\sum_{s_k} \alpha(s_k) \odot \beta(s_k) \odot \chi(s_k)}. \tag{S22}$$

(Normalizations can also be performed at any intermediate stage of the message calculations, and this is often done for numerical stability.)

4 Factor Graph EM algorithm

The message-passing algorithm given in Section 3 requires knowledge of the system parameters, i.e., the state transition probability matrix Q, the noise variance σ^2, and the current values I_{s_k}. Here we derive a factor graph EM algorithm to obtain these estimates; this algorithm is intended for use alongside the sum-product family of inference algorithms.

4.1 Estimating Q

First consider the estimation of Q. The factor graph EM algorithm proceeds iteratively in two steps. In the E-step, we calculate a function $\Phi(Q; \bar{Q})$ of both the previous estimate $\bar{Q}$ and the actual parameter value Q; then in the M-step, we maximize $\Phi(Q; \bar{Q})$ with respect to Q. The maximizing value becomes the value of $\bar{Q}$ in the next E-step, and so on until the desired number of iterations is reached. One E-step and one M-step constitutes one iteration of the EM algorithm.

With observations y, hidden states s, matrix Q of parameters (i.e. the state transition probability matrix), and estimate $\bar{Q}$ of the parameters, we have

$$\Phi(Q; \bar{Q}) = E_{\bar{Q}}\Big[\log p(y, s; Q) \mid y\Big] \tag{S23}$$

where the subscript $\bar{Q}$ indicates that the expectation is taken while setting $Q = \bar{Q}$. From (S13) we can write

$$
\begin{aligned}
&E_{\bar{Q}}\Big[\log p(y, s; Q) \mid y\Big] \\
&= E_{\bar{Q}}\left[\log \prod_{k=1}^{n} p(y_k \mid s_k) p(s_k \mid s_{k-1}; Q) \mid y\right] &\text{(S24)} \\
&= \sum_{k=1}^{n} E_{\bar{Q}}\Big[\log p(y_k \mid s_k) \mid y\Big] + \sum_{k=1}^{n} E_{\bar{Q}}\Big[\log p(s_k \mid s_{k-1}; Q) \mid y\Big]. &\text{(S25)}
\end{aligned}
$$

The first term in (S25) is constant with respect to Q and is therefore not important for the rest of the derivation, so we will absorb it into a constant C. Now we have

$$
\begin{aligned}
&E_{\bar{Q}}\Big[\log p(y, s; Q) \mid y\Big] \\
&= \sum_{k=1}^{n} E_{\bar{Q}}\Big[\log p(s_k \mid s_{k-1}; Q) \mid y\Big] + C &\text{(S26)} \\
&= \sum_{k=1}^{n} \sum_{s_k, s_{k-1}} p(s_k, s_{k-1} \mid y; \bar{Q}) \log p(s_k \mid s_{k-1}; Q) + C. &\text{(S27)}
\end{aligned}
$$

The term $p(s_k, s_{k-1} \mid y; \bar{Q})$ can be obtained directly from the sum-product algorithm, as the (normalized) product of all messages incident to the factor node $p(s_k \mid s_{k-1})$, setting $Q = \bar{Q}$ throughout the factor graph. That is, forming a $|\mathcal{S}| \times |\mathcal{S}|$ matrix $M^{(k)} = [M_{ij}^{(k)}]$, where

$$
M^{(k)} = \operatorname{diag}(\alpha^{+}(s_{k-1}))\bar{Q}\operatorname{diag}(\beta^{-}(s_k)), \qquad \text{(S28)}
$$

we have that $M_{ij}^{(k)} = p(s_k = j, s_{k-1} = i \mid y; \bar{Q})$, i.e., we can read the values of $p(s_k, s_{k-1} \mid y; \bar{Q})$ from $M^{(k)}$. Finally, returning to (S27),

$$
\begin{aligned}
&E_{\bar{Q}}\Big[\log p(y, s; Q) \mid y\Big] \\
&= \sum_{k=1}^{n} \sum_{s_k, s_{k-1} = (j,i)} M_{ij}^{(k)} \log Q_{ij} + C. &\text{(S29)}
\end{aligned}
$$

Calculation of (S29) constitutes the E-step of the EM algorithm.

The M-step is performed as follows. Recall that Q is row-stochastic, so for constant i, Q_{ij} forms a probability mass function in j. Now (S29) can be

rewritten

$$
\begin{aligned}
&E_{\bar{Q}}\Big[\log p(y, s; Q) \mid y\Big] \\
&= \sum_{s_{k-1}} \sum_{s_k} \sum_{k=1}^{n} M_{ij}^{(k)} \log Q_{ij} + C && \text{(S30)} \\
&= \sum_{s_{k-1}} Z_i \sum_{s_k} \frac{M_{ij}}{Z_i} \log Q_{ij}, && \text{(S31)}
\end{aligned}
$$

where

$$
M_{ij} = \sum_{k=1}^{n} M_{ij}^{(k)} \tag{S32}
$$

$$
Z_i = \sum_{s_k} M_{ij} \tag{S33}
$$

Each inner sum of the form $\sum_{s_k} \frac{M_{ij}}{Z_i} \log Q_{ij}$ is maximized by setting

$$
\bar{Q}_{ij}^{+} = \frac{M_{ij}}{Z_i}. \tag{S34}
$$

Thus, the M-step is accomplished by forming the matrix $\bar{Q}^{+} = [\bar{Q}_{ij}^{+}]$, with $\bar{Q}_{ij}^{+}$ given by (S34). Finally, in the next iteration, $\bar{Q}^{+}$ is fed back to the E-step as $\bar{Q}$; if no more iterations are desired, then $\bar{Q}^{+}$ is the final estimate.

4.2 Estimating σ^2

Here we derive the estimation of σ^2, which occurs simultaneously with the estimation of Q. Modifying (S25), we can write

$$
\begin{aligned}
&E_{\bar{Q},\bar{\sigma}^2}\Big[\log p(y, s; Q, \sigma^2)\Big] \\
&= \sum_{k=1}^{n} E_{\bar{Q},\bar{\sigma}^2}\Big[\log p(y_k \mid s_k; \sigma^2) \mid y\Big] + \sum_{k=1}^{n} E_{\bar{Q},\bar{\sigma}^2}\Big[\log p(s_k \mid s_{k-1}; Q) \mid y\Big]. && \text{(S35)}
\end{aligned}
$$

In (S35), note that only the first term is a function of σ^2, and only the second term is a function of Q. Thus, the E- and M-steps with respect to each parameter can be performed independently. (For Q these steps are described in detail in the previous section.)

Estimation of σ^2 for additive Gaussian noise models such as (5) is a frequently-used application of the EM algorithm, but we give the derivation here for completeness. Starting with the first term in (S35), we can write

$$\sum_{k=1}^{n} E_{\bar{Q},\bar{\sigma}^2}\Big[\log p(y_k \mid s_k;\sigma^2) \mid y\Big] = \sum_{k=1}^{n}\sum_{s_k} p(s_k \mid y;\bar{Q},\bar{\sigma}^2)\log p(y_k \mid s_k;\sigma^2) \tag{S36}$$

The term $p(s_k \mid y;\bar{Q},\bar{\sigma}^2)$ is obtained from the sum-product algorithm, and is the *a posteriori* probability of s_k given y, obtained from (S22). Meanwhile, letting I_{s_k} represent the current flowing through the patch clamp in each state s_k, from (5) we have

$$\log p(y_k \mid s_k;\sigma^2) = -\frac{1}{2}\log 2\pi\sigma^2 - \frac{(y_k - I_{s_k})^2}{2\sigma^2}. \tag{S37}$$

Let

$$W = \frac{1}{n}\sum_{k=1}^{n}\sum_{s_k}(y_k - I_{s_k})^2 p(s_k \mid y;\bar{Q},\bar{\sigma}^2). \tag{S38}$$

Then (S36) becomes

$$E_{\bar{Q},\bar{\sigma}^2}\Big[\log p(y_k \mid s_k;\sigma^2) \mid y\Big] = -n\frac{1}{2}\log 2\pi\sigma^2 - n\frac{W}{2\sigma^2}. \tag{S39}$$

Calculation of (S39) and (S29) constitute the E-step of this EM algorithm.

In the M-step, it can be shown that the maximizing value of σ^2 in (S39) is

$$\sigma^2 = W. \tag{S40}$$

Setting σ^2 as in (S40) and Q_{ij} as in (S34) completes the M-step of this EM algorithm.

5 Estimating I_{s_k}

Physical patch clamp measurements might not be calibrated, in which case I_{s_k} is an unknown parameter of the system. In this case, I_{s_k} can also be estimated by the EM algorithm: let $I_{\mathcal{O}}$ and $I_{\mathcal{C}}$ represent the new parameters, i.e. the current while the channel is open and closed, respectively.

In this algorithm, starting with (S37), the E-step is still given by (S39), but W is a function of the parameters I_O and I_C. In the M-step, we maximize (S37) with respect to $I_{\mathcal{O}}$ and $I_{\mathcal{C}}$. It can be shown that

$$\bar{I}_{\mathcal{O}} = \frac{\sum_{k=1}^{n} y_k \Pr(s_k \in \mathcal{O})}{\sum_{k=1}^{n} \Pr(s_k \in \mathcal{O})} \tag{S41}$$

$$\bar{I}_{\mathcal{C}} = \frac{\sum_{k=1}^{n} y_k \Pr(s_k \in \mathcal{C})}{\sum_{k=1}^{n} \Pr(s_k \in \mathcal{C})} \tag{S42}$$

maximizes (S39) for $I_{\mathcal{O}}$ and $I_{\mathcal{C}}$, where $\mathcal{O}$ is the set of open states, and $\Pr(s_k \in \mathcal{O}) = \sum_{s_k \in \mathcal{O}} p(s_k \mid y)$ is the posterior probability that s_k is in the open state (similarly for $\Pr(s_k \in \mathcal{C})$, where $\mathcal{C}$ is the set of closed states, replacing $\mathcal{O}$ with $\mathcal{C}$ everywhere).

For convenience, we employ an M-step that maximizes with respect to I_{s_k} first (keeping the previous estimate of σ^2), and subsequently maximizes with respect σ^2. That is, in (S38), when obtaining $\bar{\sigma}^2$ we first obtain $\bar{I}_{\mathcal{O}}$ and $\bar{I}_{\mathcal{C}}$ using the above equations, and then fix I_{s_k} at the appropriate value when calculating W. This is strictly called a *generalized EM algorithm* (GEM) and retains the EM algorithm's property of monotonically increasing likelihood [2].

6 Results for K1250A mutant

In Figures S3 and S4, we reproduce Figures 2 and 3 (respectively) from the main paper, but using the K1250A mutant of CFTR. We use the same rate matrix R as in Table 1 of the main paper, with two modifications: $R_{\mathrm{C2}\to\mathrm{O1}} = 0.2$ and $R_{\mathrm{O2}\to\mathrm{C3}} = 0.1$ (changed from original values of 4.9 and 3.0, respectively). These parameter changes reflect physiological and statistical properties of K1250A observed in [3]. Compared with the wild type, we observe that the closing rate is an order of magnitude smaller and that the ratio of nonpermissive to total closings is approximately the same, as expected. The detection performance is similar in terms of probability of false alarm, but significantly improved in terms of probability of missed detection. However, given the rarity of closings, the variance observed in these simulations is relatively high.

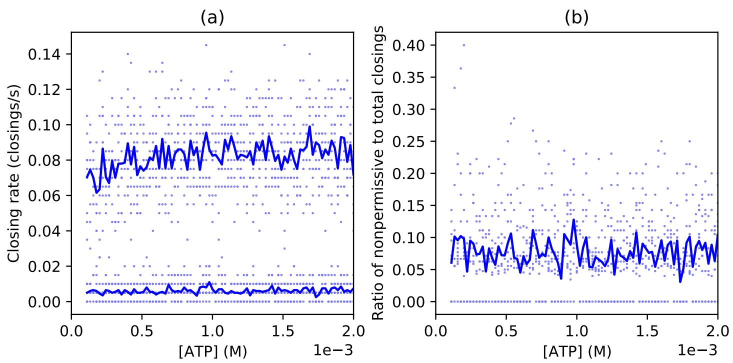

Figure S3: Prevalence of nonpermissive closings for the K1250A mutant. *Subfigure (a)*: Rate of all channel closings (top line) and nonpermissive closings (bottom line) versus ATP concentration. *Subfigure (b):* Ratio of nonpermissive to total closings versus ATP concentration. Dots represent the outcomes of each simulation run, lines represent mean value at each concentration. Sampling rate = 100 Hz, rate parameters as described above.

References

[1] Frank R Kschischang, Brendan J Frey, and H-A Loeliger. Factor graphs and the sum-product algorithm. *IEEE Trans. Inf. Theory*, 47(2):498–519, 2001.

[2] Arthur P Dempster, Nan M Laird, and Donald B Rubin. Maximum likelihood from incomplete data via the EM algorithm. *J. R. Stat. Soc. Series B Stat. Methodol.*, 39(1):1–22, 1977.

[3] Matthew D Fuller, Zhi-Ren Zhang, Guiying Cui, and Nael A McCarty. The block of CFTR by scorpion venom is state-dependent. *Biophys. J.*, 89(6):3960–3975, 2005.

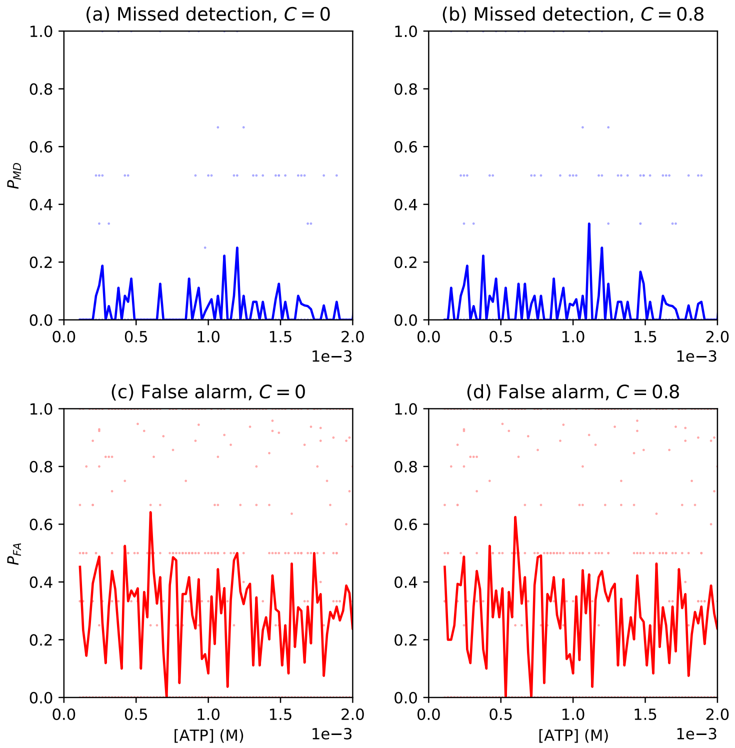

Figure S4: Missed detection and false alarm probabilities for the K1250A mutant. *Subfigures (a), (b):* Missed detection probability versus ATP concentration for confidence threshold $C = 0$ and $C = 0.8$, respectively. *Subfigures (c), (d):* False alarm probability versus ATP concentration for $C = 0$ and $C = 0.8$, respectively. Dots represent each simulation run, while lines represent the average at each concentration. Sampling rate = 100 Hz, 20000 samples, 400 EM iterations, $\sigma^2 = 0.02$, rate parameters as described above.
